# Opportunities of Covalent Organic Frameworks for Advanced Applications

**DOI:** 10.1002/advs.201801410

**Published:** 2018-11-12

**Authors:** Yanpei Song, Qi Sun, Briana Aguila, Shengqian Ma

**Affiliations:** ^1^ Department of Chemistry University of South Florida 4202 E Fowler Ave. Tampa FL 33620 USA

**Keywords:** covalent organic frameworks, crystalline materials, porous materials, porous polymers, task‐specific designs

## Abstract

Covalent organic frameworks (COFs) are an emerging class of functional nanostructures with intriguing properties, due to their unprecedented combination of high crystallinity, tunable pore size, large surface area, and unique molecular architecture. The range of properties characterized in COFs has rapidly expanded to include those of interest for numerous applications ranging from energy to environment. Here, a background overview is provided, consisting of a brief introduction of porous materials and the design feature of COFs. Then, recent advancements of COFs as a designer platform for a plethora of applications are emphasized together with discussions about the strategies and principles involved. Finally, challenges remaining for this type material for real applications are outlined.

## 1 Introduction

### 1.1 Porous Materials

Porous materials are identified by their pores, which include cavities, channels, or interstices. They are fundamental in a diverse range of applications, from structural materials to energy technologies.^1^ The properties of a porous material and their suitability for different applications are intimately connected, varying depending on the size, arrangement, and shape of the pores, as well as the porosity and composition of the material itself.^2^ To this end, sustained efforts are bringing scientists ever closer to the long sought after goal of being able to manipulate the compositions and structures of porous materials, as these determine the function and control accessibility to the internal surface of the porous materials. A major recent advance in this regard has been the development of modular construction, using molecular components that can be connected predictably to adjoin subunits.^3^ This has led to a sudden wealth of remarkable porous materials, including metal–organic frameworks (MOFs), held together by coordination bonds,^4^ and covalent organic frameworks (COFs), in which atoms of carbon and other light elements are bonded covalently to define open networks.^5^ Prior to these, there has been no material that is able to precisely control the structure and composition simultaneously. The modular nature of these materials allows their pore geometries and chemical functionalities to be fine‐tuned independently and thus enabling rigorous comparison to establish correlations between individual parameters, which can be leveraged to further tailor the outcomes. The environmentally benign and economically beneficial applications of porous materials in industrial catalysis, adsorption, and ion‐exchange processes drive a continuing effort into the research of their fundamental properties and manipulation of their structures and function to enhance the performance. The on demand synthesis of COFs and MOFs positions them as ideal candidates to develop structure–property relationships as well as to address the enduring challenges pertinent to energy and environmental sustainability, such as gas storage, gas separation, catalysis, environmental remediation, and chemical sensing.^6^

### 1.2 Design Features and Advantages of COFs

COF synthesis was inspired by prior advances in other classes of framework materials and designed noncovalent assemblies, in particular from the concepts used for MOF synthesis. One of the challenges of extending MOF design concepts to covalently linked polymers is that many organic functional groups, characteristically possessing high bond strengths, do not undergo dynamic exchange processes as readily as coordination complexes. As a result, linking organic molecules by covalent bonds into extended solids typically generates amorphous, disordered materials.[[qv: 5a]] The attainment of crystals was accomplished by several techniques in which a balance is struck between the kinetics and the thermodynamic reversibility of the linking reactions. This breakthrough was made by Yaghi and co‐workers when they reported the very first two COF members.^7^ To maintain reversible conditions, these COFs were synthesized in a closed pyrex tube under solvothermal conditions to ensure that H_2_O is available for bond self‐correction. A boronate ester‐linked network designated COF‐5, obtained from the condensation of 1,4‐benzenediboronic acid (BDBA) and 2,3,6,7,10,11‐hexahydroxytriphenylene (HHTP), which represents a prototypical 2D COF structure. The COF‐5 network adopts a hexagonal unit cell with the porous sheets stacked in a nearly eclipsed fashion with a 0.34 nm interlayer spacing. This structure, as well as all subsequent 2D COFs, was assigned by comparing its refined powder X‐ray diffraction (PXRD) pattern with simulated patterns of eclipsed and staggered stacking arrangements; no single‐crystal X‐ray structure of a 2D COF has been reported so far. COF‐5 exhibits permanent porosity with a Brunauer–Emmett–Teller (BET) surface area of 1590 m^2^ g^−1^, a narrow pore‐size distribution centered at 2.7 nm, and excellent thermal stability (to temperatures up to 500 to 600 °C). The other 2D COF, COF‐1, derived from the self‐condensation of BDBA was reported simultaneously with COF‐5, and remains the single example of staggered interlayer stacking. These successes have led to the expansion of COF materials to include organic units linked by strong covalent bonds such as B—O, C—N, and C—C.[[qv: 1l,m,5c,6l]] Due to the diversity of organic synthesis, the enormous possible design space available within COFs provides virtually unlimited room for imagination, allowing designed incorporation of different functionalities for numerous potential applications. In contrast to other porous materials, the control of geometry, size, and functionality of the building blocks can be used to design not only the atomic layer structure, but also the framework structure. In this context, the properties of COFs can be designed using specific structures of knots and linkers in the topology diagrams. Thus, COFs offer a combination of properties not found in other materials.

#### 1.2.1 Low Density

COFs are constructed solely from light elements that are expected to offer high gravimetric performance for guest molecule and energy storage. The density for COF‐108 is only 0.17 g cm^−3^, lower than any other crystalline solid.^8^

#### 1.2.2 Stability

COFs are connected by robust covalent bonds and thereby show enhanced stability compared to most MOFs. Recent advances in reinforcing the COF structures include providing hydrogen bonding interactions, weakening the polarity of the amine bond, introducing enol‐keto tautomerizations, or utilizing a Michael addition–elimination/benzoxazole pathway.^9^ These state‐of‐the‐art approaches to stabilize COFs result in materials that are highly stable to hydrolysis, a wide range of pHs, and reductive and oxidative environments, which has barely been demonstrated in a MOF.

#### 1.2.3 Crystallinity

COFs are crystalline, offering a strategy to position functional groups in a highly controlled and predictable manner. This establishes the structure–property relationship and enables characterization using diffraction techniques. This structural uniformity is also potentially attractive for optoelectronic devices and catalysis.

#### 1.2.4 Porosity

COFs have periodic and uniform porosity allowing for better performance in gas separation or catalysis where usually full access to the pores is required. Surface areas exceeding 3000 and 5000 m^2^ g^−1^ have been reported for 2D and 3D COFs, respectively.

#### 1.2.5 Modularity

COFs' versatile properties can be adjusted from a thoughtful selection of building units prior to synthesis. Therefore, scientists can manage the composition and the structural architecture in a crystalline and porous material with precise control over chemical functionality, density, and spatial arrangement of the active sites. Recent developed strategies, such as incorporating monomers with reduced symmetry, multiple components with differing lengths, more than one bond forming process, or using metal coordination approaches, enable the COF structures to be more sophisticatedly controlled. Depending on the desired properties and functions, COFs can be designed at three different structural levels: pore design, skeleton design, and the complementary design of pores and skeletons.^10^, ^11^ This universal control makes COFs an attractive platform for designed molecular assembly that has not yet been fully exploited.

In this review, we will highlight representative examples that leverage the above features to construct COFs with promising properties. We aim to give a concise overview about the multitude of properties of COFs and applications that have been realized so far, providing inspiration for further development and utilization of these intriguing structures. A main focus will be on insights regarding the task‐led design. Beyond assembling diverse molecular linkers in coupling reactions to generate the frameworks, additional functionality can be incorporated into COFs through postsynthetic modification as well as integration with other functional materials and these will be introduced along with their specific application. Challenges remain for this type of material pertaining to their practical applications, such as convenient methods to process COFs into useful forms and scalable synthesis, which will be introduced in the last section.

## 2 Functional Exploration and Applications

### 2.1 Gas Storage/Separations

Inspired by an escalating interest in MOFs and other porous materials for storing, separating, or sequestering technologically relevant gases,^12^ COFs were initially evaluated for these applications.^13^ COFs are comprised of lightweight elements with low density that would provide improved gravimetric storage capacity, making them excellent candidates for gas storage. Due to its importance in clean energy, great efforts have been devoted to finding materials with high hydrogen storage capacities, and COFs have also been investigated for this purpose. Theoretical studies on prototypical COFs predicted that at 77 K, up to 10.0 wt% excess H_2_ storage capacity can be achieved in COF‐105 at 80 bar.[[qv: 13a]] This value obviously outperforms representative MOFs whose H_2_ uptake values were 7.0 and 7.1 wt% for MOF‐177[[qv: 14a]] and MOF‐5,[[qv: 14b]] respectively. With respect to volumetric capacity, COF‐102 boasts the maximum excess H_2_ uptake of 40.4 g L^−1^ at 100 bar, close to the target of 45 g L^−1^ identified by the Department of Energy, ranking it among the highest performing materials in terms of gas storage capacities.[[qv: 14c]] Stimulated by these theoretical results, Furukawa and Yaghi demonstrated the first adsorption studies of H_2_, CH_4_, and CO_2_ relevant to clean energy applications in COFs.^15^ It was found that 3D COFs with medium‐sized pores, such as COF‐102 and COF‐103, showed superior hydrogen storage capacity among all reported COFs, with hydrogen uptake values of 72.4 and 70.5 mg g^−1^, respectively, at 77 K and 85 bar, rivaling some of the best MOFs and other porous materials in their uptake capacities. These results suggest that COFs give great potential in the quest for practical gas storage materials.

**Figure 1:**
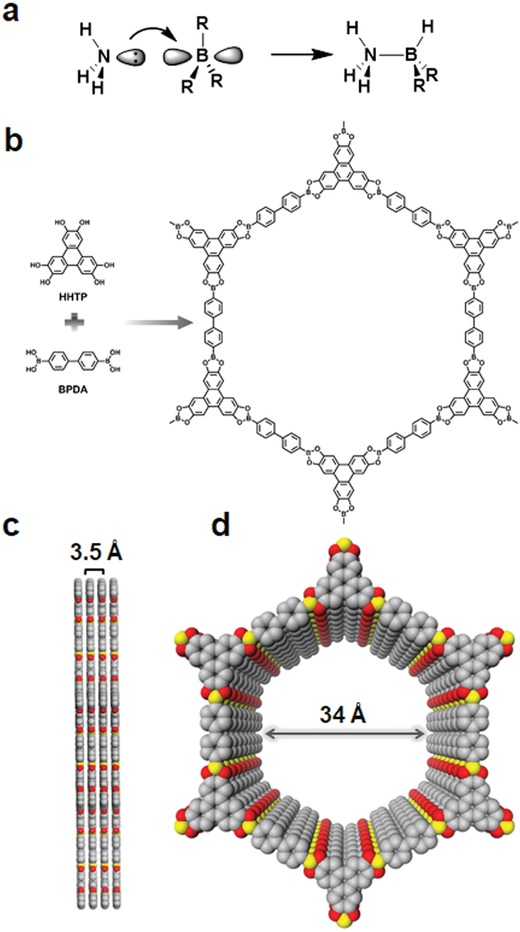
a) Ammonia‐boron (Lewis acid–base) interactions present on the surface of COF‐10. b) Schematic representation of a COF‐10 pore showing its atom connectivity and its organic building blocks. c,d) Graphic view of the eclipsed AA stacking structure of COF‐10 (yellow, B; gray, C; red, O; hydrogen is omitted for clarity). Reproduced with permission.^17^ Copyright 2010, Nature Publishing Group.

In addition to gas storage, COFs are also useful for the capture and storage of harmful gases. Due to its irreplaceable role in global agriculture and industry, ammonia is one of the largest volume chemicals on the planet, yet it is a highly toxic and corrosive gas even in small concentrations.^16^ Therefore, sorbents capable of capturing NH_3_ are of interest for industrial ammonia transportation, separation of NH_3_ from N_2_ and H_2_, personal protective equipment, as well as air remediation. Considering the high density of Lewis acidic boron atoms, present in boroxine (B_3_O_3_) or boronate ester (C_2_O_2_B) rings in some COFs, these sites provide a unique adsorbent surface ready for trapping Lewis basic gases, such as ammonia, reminiscent of the classical ammonia–borane coordinative bond (**Figure** **1**a). Given this, COF‐10 synthesized by the condensation of hexahydroxytriphenylene and biphenyldiboronic acid was chosen for evaluation (Figure 1b).^17^ The formed porous hexagonal sheets in this 2D COF are stacked in an approximately eclipsed pattern with 1D pores with diameters of 34 Å and each hexagonal ring contains 12 boron atoms as part of the five‐membered boronate ester rings (C_2_O_2_B, Figure 1c,d). An ammonia adsorption isotherm collected at 25 °C for COF‐10 showed exceptional uptake (15 mol kg^−1^), significantly outperforming the state‐of‐the‐art materials Zeolite 13× (9 mol kg^−1^) and Amberlyst 15 (11 mol kg^−1^). A noteworthy feature of COF‐10 is that the adsorbed NH_3_ can be completely removed by heating to 200 °C under a pressure of 0.1 torr and repeated adsorption/desorption cycling did not result in any significant loss of NH_3_ uptake capacity for at least three cycles. However, broadening and a decrease in intensity of the PXRD patterns of COF‐10 subsequent to each cycle indicated that the ammonia adsorption resulted in turbostratic structural disorder and increasing disorder in packing between layers during cycling. This was further evidenced by the decreasing mesoporous character as reflected by its gradually lowering N_2_ uptake capacity. To eliminate the issue of irreversibly disordering lattice packing during cycling, using Lewis acidic 3D COFs might improve the long‐term performance, given that their entire frameworks are connected by covalent bonds.

Recent studies have illuminated the impact of open metal sites on the ammonia capacity of MOFs, but a vast majority of MOFs cannot survive under ammonia exposure. To address this issue, the advantages of both MOFs and COFs can be combined, as the covalent bond constructed backbone provides stability, while the introduction of metal ions with accessible coordination sites confers a high affinity for ammonia. In this context, Yang et al. reported functionalization of an imine‐linked 2D COF with carboxylic acid groups decorating the pore walls with a controlled amount by using a multicomponent condensation strategy.^18^ The material with no acidic groups has an NH_3_ uptake capacity of 6.85 mmol g^−1^ at 1 bar, 298 K, which increases nearly 50% to 9.34 mmol g^−1^ for the material with 17% acid functionalization. Furthermore, the authors incorporated Ca, Sr, and Mn divalent cations to the pore surface by treatment of the best performing 17% acid‐functionalized COF with corresponding metal solutions. The ammonia uptake capacity was significantly enhanced in all three cases, particularly for the strontium incorporated material, with an equilibrium ammonia capacity of 14.30 mmol g^−1^ under identical conditions. These results provided a way to increase the interaction strength between the COF materials and the target molecules and thus improve the uptake capacity and selectivity.

**Figure 2:**
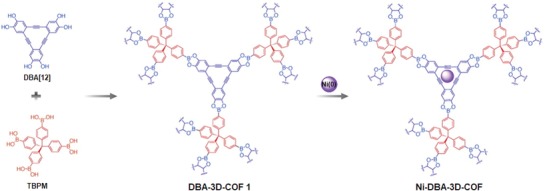
Synthesis of DBA‐3D‐COF 1 using TBPM and DBA[12] units followed by metalation with Ni(COD)_2_ to produce Ni‐DBA‐3D‐COF. Reproduced with permission.^19^ Copyright 2016, American Chemical Society.

Given this, the utilization of building units that are capable of forming strong metal complexes is preferred for designing high performance gas adsorption materials. By virtue of the unique planar structure and flexible electron donation property of dehydrobenzoannulene (DBA), McGrier and co‐workers demonstrated the synthesis and metalation of a mesoporous 3D COF containing DBA.^19^ The target COF, DBA‐3D‐COF 1, was synthesized by reacting DBA[12] (see **Figure** **2**) with tetrahedral tetra‐(4‐dihydroxyborylphenyl)methane (TBPM) with a BET surface area of 5083 m^2^ g^−1^, the highest value reported to date for a COF. Metalation was readily achieved by treatment of the COF with Ni(COD)_2_ toluene solution, yielding Ni‐DBA‐3D‐COF with a retained surface area (4763 m^2^ g^−1^, Figure 2). Ethane/ethylene sorption isotherms for DBA‐3D‐COF 1 and Ni‐DBA‐3D‐COF revealed that a moderate increase of 0.07 mmol for ethane and 0.13 mmol for ethylene at 295 K were achieved. Despite the slight increase, this proof‐of‐principle is important given that it increases the prospect of using metalated COFs for related applications. In addition to this, designing a material with suitable pore size is an alternative way to enhance the selectivity. For example, Zhu and co‐workers synthesized a new 3D microporous COF (MCOF‐1) with a uniform pore size of 0.64 nm by condensation of the TBPM and 1,2,4,5‐tetrahydroxybenzene.^20^ With the appropriate pore size and high surface area, this material exhibited excellent C_2_H_6_/CH_4_ and C_2_H_4_/CH_4_ adsorption selectivity, surpassing the representative porous adsorbents and positioning it among the best materials for separating C_2_ hydrocarbons from CH_4_.

The development of effective technologies or strategies that can efficiently capture CO_2_ and mitigate this environmental dilemma is an urgent task. Since the first report on CO_2_ adsorption of COFs in 2009, great efforts have been made to improve the CO_2_ capture performance of these materials.[[qv: 6k]] Despite great progress achieved, there is still room to be improved. The performance of COFs as CO_2_ sorbents in terms of both uptake capacity and selectivity are not comparable with their analogues, MOFs, which is primarily due to their binding site deficiency and less suitable pore sizes. The following are some representative works to address these challenges.

With the consideration that azine‐linked COFs not only possess exceptional stability, but also can be easily predetermined to make smaller pore sizes due to the short structural length of the azine unit, Liu and co‐workers synthesized an azine‐linked COF, ACOF‐1, by Schiff base reactions between hydrazine and 1,3,5‐triformylbenzene.^21^ Due to its high surface area (1176 m^2^ g^−1^), large pore volume, and relatively small pore size (0.94 nm) as well as abundant nitrogen sites on the pore wall, ACOF‐1 exhibited a high CO_2_ uptake capacity of 177 mg g^−1^_,_ superior to many representative COFs, even those with higher surface area.

**Figure 3:**
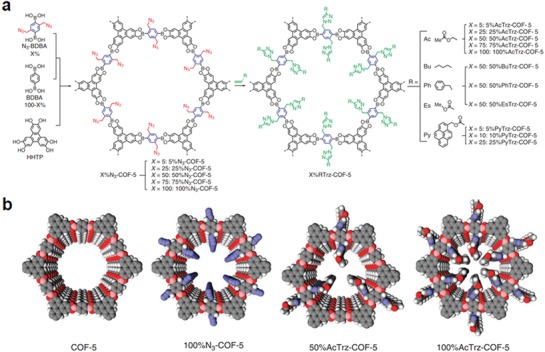
a) The scheme shows a general strategy for the surface engineering of COFs through the combination of condensation reactions and click chemistry. In the first step, COFs bearing azide units on the walls are synthesized by the condensation reaction of HHTP with azide‐appended benzene diboronic acid (N_3_‐BDBA) and benzene diboronic acid (BDBA) in a designated molar ratio (*X* = 0–100%). The content of N_3_‐appended wall units is tunable from 0 to 100%, depending on the molar ratio of N_3_‐BDBA to BDBA. Five members of *X*%N_3_^−^ COF‐5 with different contents (*X* = 5, 25, 50, 75, and 100) were synthesized. The structure of 100%N_3_‐COF‐5 is shown in the figure, with all of the walls occupied by the N_3_‐appended phenylene units. In the next step, the azide groups on the COF walls are clicked with alkynes to anchor various organic groups onto the walls of COFs (*X*%RTrz‐COF‐5). The density of R surface groups on the walls is determined by the azide content in *X*%N_3_‐COF‐5. b) Graphical representation of COF‐5 upon surface engineering, which leads to the functionalization of organic groups on the walls. The composition and density of the organic groups on the walls are controllable. Adapted with permission.^23^ Copyright 2011, Nature Publishing Group.

Given the importance of pore surface properties on the sorption behavior toward specific gas and thereby the capacity and selectivity, tailor‐made interface of COFs would offer numerous possibilities for gas storage and separation.^22^ Jiang and co‐workers reported the first example with this respect. Specifically, to introduce various functionalities onto the pore wall and to precisely control the composition, density, and functionalities of organic groups anchored onto the pore walls, a three‐component topological design was developed. HHTP was cocrystallized with an azide appended N_3_‐BDBA and 1,4‐benzenediboronic acid at various rations, yielding a series of isostructures of COF‐5 with controllable azide densities (*X*%N_3_‐COF‐5).^23^ The azide units on the COF walls then underwent a quantitative click reaction with alkynes to form triazole‐linked groups on the wall surfaces (**Figure** **3**). Several cycloaddition partners and functionalization densities demonstrate the versatility of this strategy to introduce external functionality throughout the porous crystals. To investigate the role of these introduced functionalities, the gas‐sorption selectivity of CO_2_ over N_2_ for the surface‐engineered COF‐5 was compared with that for the pristine COF‐5. It was shown that both the type and the content of functionalities have a great effect on their adsorption. For example, a fivefold increased selectivity in comparison with COF‐5 was found in 25%PyTrz‐COF‐5 and 100%AcTrz‐COF‐5 showed a 16‐fold increase in selectivity relative to that of COF‐5. Given the great improvement, this strategy opened a new avenue to improve the gas adsorption/separation performance of COFs. Later, they extended the pore surface engineering strategy to imine‐linked porphyrin COFs to enhance their affinity toward CO_2_.^24^ Considering that material's gas adsorption performance is usually determined by its porosity and the functionalities therein, a similar three‐component reaction system was used to adjust the content of ethynyl groups and thereby to achieve the trade‐off between the porosity and the content of functionalities in the resultant COFs to screen the best performance CO_2_ adsorbents. Twenty COF materials with different types or density of functionalities were synthesized by Cu catalyzed click reactions between the ethynyl groups in the COFs and various azide compounds. Due to the strong interactions between the amino groups and CO_2_ by the formation of acid–base pairs, a significant enhancement in CO_2_ adsorption was observed for the amine functionalized COFs, even tripling that of the pristine COF.

### 2.2 Catalysis

In view of the continuous environmental and economic challenges in the world, there is a pressing need to develop a more sustainable chemical industry by implementing efficient chemical transformations. This can be done by developing new catalytic materials to improve the outputs and thereby lower the overall process costs. The use of porous materials as heterogeneous catalysts is of great interest because they offer the advantages of conventional heterogeneous catalysis combined with greater accessibility of active sites.^25^ Beyond the traditional porous material, the great tunability of COFs gives this new type of porous material high potential in catalysis, which display advantageous features of both molecular and heterogeneous systems. The construction with molecular building blocks enables precise manipulation of the spatial arrangement of catalytic centers within the predetermined COF structure, allowing for rational design at the molecular level.

**Figure 4:**
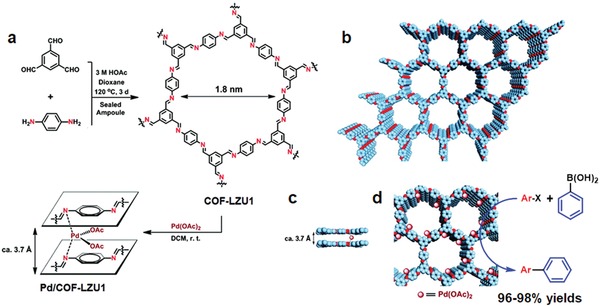
a) Construction of COF‐LZU1 and Pd/COF‐LZU1. Schematic representation of the synthesis of COF‐LZU1 and Pd/COF‐LZU1 materials. b–d) Proposed structures of COF‐LZU1and Pd/COF‐LZU1 simulated with a 2D eclipsed layered‐sheet arrangement. C: blue, N: red, brown spheres represent the incorporated Pd(OAc)_2_, and H atoms are omitted for clarity. Reproduced with permission.^26^ Copyright 2011, American Chemical Society.

Wang and co‐workers reported the first application of COFs as an ideal scaffold for catalysis. A 2D imine‐linked framework, COF‐LZU1, by condensing 1,3,5‐triformylbenzene (TFB) and 1,4‐diaminobenzene, was newly designed to meet the prerequisite as a catalyst, that is, stability and the ability to incorporate catalytic active sites.^26^ This COF adopts a nearly eclipsed layered crystal with 1.8 nm wide pores, and is hypothesized to bind metal ions through bidentate coordination to proximal N atoms in adjacent layers. To load Pd species, COF‐LZU1 was treated with a Pd(OAc)_2_ solution resulting in only minor changes to its PXRD pattern (**Figure** **4**). The obtained Pd‐loaded COF showed high activity in the Suzuki–Miyaura cross‐coupling reaction with broad scope of the reactants, achieving high yields (96–98%) at low catalyst loadings (less than 1 mol% Pd). More significantly, the insoluble COFs were readily recovered and recycled at least four times without loss of activity. This study demonstrates a promising future for COF‐based catalysts to combine specific metal‐coordination environments and the operational simplicity of heterogeneous catalysis, though further improvements in stability are necessary.

Gaining enlightenment from this work, Gao and co‐workers nicely demonstrated that COFs provide an intriguing platform for precise control of the number and position of the catalytic metal ions. To show this, a series of 2D COFs containing two different types of nitrogen ligands, namely imine and bipyridine, with controllable content were synthesized by a three‐component condensation system.^27^ Using a programmed synthetic procedure, two metal complexes, Rh(COD)Cl and Pd(OAc)_2_, with different size can be selectively coordinated with the two nitrogen ligands in the COFs. The large and rigid Rh(COD)Cl molecules which do not fit into the space between adjacent COF sheets were first introduced into the COFs to coordinate with bipyridine moieties and then the smaller and more flexible Pd(OAc)_2_ molecules were incorporated to coordinate with the residual bipyridine moieties, if have, or amine moieties. The bimetallically docked COFs showed excellent activity in a one‐pot addition–oxidation cascade reaction.

**Figure 5:**
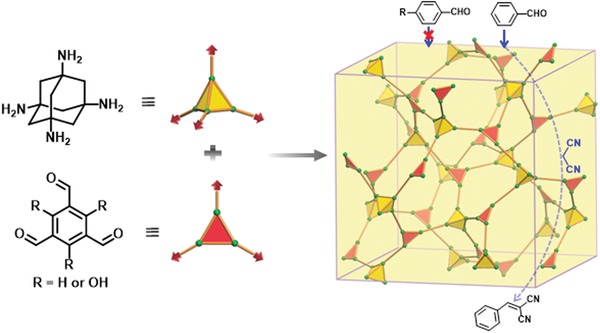
Schematic representation of the strategy for preparing a 3D microporous base‐functionalized COF by condensation of 1,3,5,7‐tetraaminoadamantane (TAA) and 1,3,5‐ triformylbenzene or triformylphloroglucinol and its application in size selective Knoevenagel condensation reactions. Reproduced with permission.^28^ Copyright 2014, John Wiley and Sons.

With the knowledge of the basicity of an amine bond formed by the alkyl amine and aldehyde species as well as the microporosity of 3D COFs, Yan and co‐workers designed and synthesized two new 3D COFs by condensing a tetrahedral alkyl amine, 1,3,5,7‐tetraaminoadamantane (TAA) with triangular building units TFB or triformylphloroglucinol (TFP) for their use as base catalysts (**Figure** **5**).^28^ The obtained materials exhibited remarkable activity in the Knoevenagel condensation reaction of benzaldehyde and malononitrile (>96%). Importantly, due to the microporous cavities, excellent substrate selectivity imparted by the pore structure was observed, as revealed by the fact that aldehyde reactants with dimensions larger than the pore aperture gave very low yields (<5%).

Given the important role of acid catalysis in industry, the development of efficient solid acid materials is highly desired. Zhao and co‐workers reported the de novo synthesis of a sulfonated COF (TFP‐DABA) by condensation between 1,3,5‐ TFP and 2,5‐diaminobenzenesulfonic acid (DABA).^29^ The resultant TFP‐DABA was initially studied as a solid acid catalyst in fructose dehydration to 5‐hydroxymethylfurfural (HMF). Due to the high density of accessible acid groups in TFP‐DABA, it displayed remarkable catalytic activity and selectivity, reaching the fructose conversion and HMF yield of 100 and 97%, respectively, within 1 h. In addition, with the assistance of KBr, this material can efficiently catalyze fructose into 2,5‐diformylfuran with full conversion and 65% selectivity. Moreover, this catalyst was stable as verified by its excellent recyclability.

Employing CO_2_ as an abundant, inexpensive, and nontoxic C1 source could contribute to the sustainable use of natural resources.^30^ Knowing that a catalyst bearing hydrogen bond donors can accelerate the cycloaddition reaction between CO_2_ and epoxides, a COF material (2,3‐DhaTph) containing catechol as the hydrogen bond donor was constructed by Schiff base reactions of 5,10,15,20‐tetrakis(4‐aminophenyl)‐21H,23H‐porphine (Tph) and 2,3‐dihydroxyterephthalaldehyde (Dha). The resultant COF showed excellent activity toward cyclic carbonates and oxazolidinones with the assistance of tetrabutylammonium iodide in metal free conditions.^31^

Considering that quaternary ammonium salts have been proven to be efficient catalysts for CO_2_ transformations, to install ionic moieties, the COFs with different phenol content on their channel walls were synthesized as intermediates by three‐component condensation of 4,4′,4″,4′″‐(pyrene‐1,3,6,8‐tetrayl) tetraaniline (PyTTA) with 2,5‐dihydroxyterephthalaldehyde (DHPA) and 1,4‐phthalaldehyde.^32^ The resultant COFs were nominated as [HO]*_X_*_%_‐Py‐COFs (*X* = 0, 25, 50, 75, 100), where X% represents the molar percentage of DHPA used in the dialdehyde blend. Through the Williamson ether reaction between (2‐bromoethyl)triethylammonium bromide and phenol group in [HO]*_X_*_%_‐Py‐COFs, ionic moieties were then immobilized onto the COFs named as [Et_4_NBr]*_X_*_%_‐Py‐COF. Considering the trade‐off of density of ionic moieties, as well as the crystallinity and surface of the functionalized COFs, [Et_4_NBr]_50%_‐Py‐COF was chosen as a representative sample for detailed catalytic evaluation. Under very mild conditions, at 30 °C and 1 bar CO_2_, it can efficiently catalyze the formylation of various amines with CO_2_ in the presence of phenylsilane (PhSiH_3_) with good recyclability.

**Figure 6:**
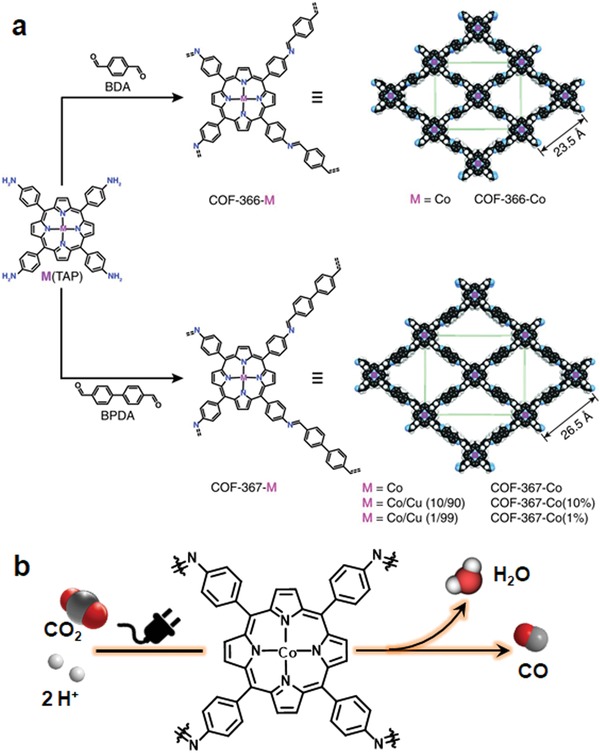
a) Design and synthesis of metalloporphyrin‐derived 2D covalent organic frameworks. Adapted with permission.^33^ Copyright 2015, American Association for the Advancement of Science. b) Schematic illustration of cobalt porphyrin‐base COFs for catalytic CO_2_ reduction in water. Reproduced with permission.^34^ Copyright 2018, American Chemical Society.

Yaghi and co‐workers demonstrated an elegant example of modular optimization of COFs to prepare a high performance catalytic material as demonstrated by aqueous electrochemical reduction of CO_2_ to CO. Initially, a model COF material (COF‐366‐Co) by the imine condensation of 5,10,15,20‐tetrakis(4‐aminophenyl)porphinato]cobalt [Co(TAP)] with 1,4‐benzenedicarboxaldehyde (BDA) was synthesized for electrocatalysis studies (**Figure** **6**). It was found that COF‐366‐Co exhibited high selectivity over competing proton reduction with a Faradaic efficiency for CO of 90% as well as excellent durability with stable outputs for at least 24 h, giving a corresponding turnover number (TON) of 34 000, far outperforming the molecular Co(TAP) (TON ≈ 8300).^33^ More significantly, COF‐366‐Co showed greater than 10% enhancement in CO_2_ to proton selectivity over the molecular Co(TAP). Considering that a larger pore size would allow for higher capacity of carbon dioxide adsorption inside the framework as well as more efficient exposure of the electroactive sites to the reactants, a modular reticular approach was used to further improve the performance of COF‐366‐Co. To expand the pore size, biphenyl‐4,4′‐dicarboxaldehyde was used as the strut instead of BDA to condense with Co(TAP) for synthesizing the COF. As expected, this expanded COF exhibited improved catalytic efficiency over COF‐366‐Co, giving a TON value of 48 000 after 24 h. Taking advantage of the tunable synthesis, to further optimize the performance, the authors introduced building‐block heterogeneity through a multivariate strategy to maximum the utilization efficiency of active sites. To achieve this, catalytically inactive isostructural copper porphyrin units were partly replaced by Co(TAP) to dilute its concentration with the purpose of increasing the proportion of the active sites exposed to the reactant and thereby improve the turnover frequency on a per‐cobalt basis. Accordingly, two bimetallic COF‐367 derivatives with the Co proportion of 10% and 1% in all metal sites were synthesized named as COF‐367‐Co(10%) and COF‐367‐Co(1%), respectively. A substantial improvement with each tenfold dilution of cobalt loading was observed. Specifically, at the beginning 4 h, COF‐367‐Co, COF‐367‐Co(10%), and COF‐367‐Co(1%) afforded the turnover frequency (TOF) values of 1900, 4400, and 9400, respectively. To further enhance the active sites accessibility, COF‐366‐Co thin films were in situ grown on glassy carbon to improve electrochemical contact and thereby the catalytic efficiency. Under the same electrolysis conditions, a seven times increase in terms of TOF was afforded compared with the corresponding powder sample. Subsequently, the performance of the catalysts was further systematically tuned by modification of the reticular structure and the current density for CO formation increases from 45 mA mg^−1^ for COF‐366‐Co, to 46 mA mg^−1^ for COF‐366‐(OMe)_2_‐Co, and up to 65 mA mg^−1^ for COF‐366‐F‐Co.^34^

**Figure 7:**
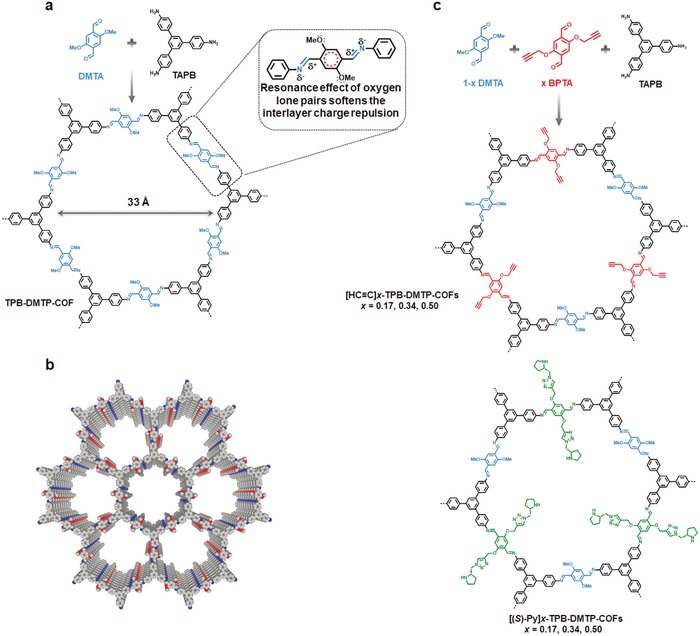
Synthesis and structure of stable crystalline porous COFs. a) Synthesis of TPB‐DMTP‐COF through the condensation of DMTA (blue) and TAPB (black). Inset: The structure of the edge units of TPB‐DMTP‐COF and the resonance effect of the oxygen lone pairs that weaken the polarization of the C=N bonds and soften the interlayer repulsion in the COF. b) Graphic view of TPB‐DMTP‐COF (red, O; blue, N; grey, C; hydrogen is omitted for clarity). c) Synthesis of chiral COFs ([(S)‐Py]*x*‐TPB‐DMTP‐COFs, *x* = 0.17, 0.34, and 0.50; blue, DMTA; black, TAPB; red, BPTA; green, (*S*)‐Py sites) via channel‐wall engineering using a three‐component condensation followed by a click reaction. Adapted with permission.^36^ Copyright 2015, Nature Publishing Group.

The development of robust heterogeneous chiral catalysts that can perform asymmetric reactions with high efficiency and superb enantiomeric selectivity is of great importance.^35^ A material that combines the features of stability, crystallinity, and porosity is highly sought after for the design of efficient catalysts. Due to the polarization of the C=N bond, this causes electrostatic repulsion and destabilizes the layered structure of the 2D COF, thereby leading to low structural stability. To tackle this challenge, Jiang and co‐workers proposed a strategy to weaken the polarization influence by delocalizing the lone pairs of electron donating groups over the positively charged phenyl rings through resonance effects as demonstrated by using a methoxy group substituted monomer (**Figure** **7**a,b).^36^ Accordingly, a crystalline porous COF (TPB‐DMTP‐COF) that is stable against water, strong acids (12 m HCl), and strong bases (14 m NaOH), was synthesized by condensation of dimethoxyterephthaldehyde (DMTA) and 1,3,5‐tri‐(4‐aminophenyl)benzene (DMTP) under solvothermal conditions. In addition to the exceptional stability, this material also possesses high crystallinity, giving a BET surface area of 2105 m^2^ g^−1^, which is among the highest for 2D COFs, combined with a very narrow pore size distribution centered at 3.3 nm. All of these properties exceed the state‐of‐the‐art approaches to stabilize COFs including the introduction of enol‐keto tautomerizations or hydrogen bonding interactions to the COF skeletons. Encouraged by these intriguing properties, the authors further explored its application in chiral catalysis. To introduce chiral centers and to adjust the density of active sites, a three‐component condensation system with 2,5‐bis(2‐propynyloxy)terephthalaldehyde (BPTA) and DMTA as edge units was employed to prepare the intermediate [HC≡C]*x*‐TPB‐DMTP‐COFs (Figure 7c). Following a quantitative azide‐ethynyl click reaction, a chiral organocatalytic site of pyrrolidine was anchored. The resultant catalysts are also stable to aqueous HCl (12 m) and NaOH (14 m) solutions. Catalytic evolution results revealed that the resultant catalysts exhibited excellent activities and enantioselective selectivities as well as broad substrate tolerance. Due to its large pore size, it permits the face‐on attack by nitrostyrenes to the catalytic sites and thereby the high activity, placing them among the most efficient Michael reactions catalysts reported thus far.

Compared with postsynthetic modification, the utilization of de novo approach is expected to offer better control with respect to the grafting degree and homogeneity of functionalities. Wang and co‐workers reported the first example of the direct construction of chiral COFs from chiral building blocks.^37^ Specifically, condensation of a judiciously designed chiral unit of (*S*)‐4,4′‐(2‐(pyrrolidin‐2‐yl)‐1H‐benzo[d]imidazole‐4,7‐diyl)dianiline with TFB or TFP yielded two chiral COFs named as LZU‐72 and LZU‐76, respectively. Both of them showed high crystallinity and relatively high surface areas of 1114 and 748 m^2^ g^−1^, respectively. Due to the robustness of β‐ketoenamine linkages in LZU‐76 against acidic conditions, it was then chosen for catalytic tests. In the asymmetric aldol reaction, LZU‐76 can efficiently catalyze the reactions between a variety of aromatic aldehydes and acetone in the presence of trifluoroacetic acid with competitive enantioselectivity in relation to its homogeneous counterpart.

Cui and co‐workers also described the preparation and catalytic behavior of a series of 2D COFs bearing chiral organocatalysts such as L‐proline and L‐imidazolidine.^38^ Notably, the multiple component strategy used for the construction of chiral COFs not only allows for adjustment of the concentration and spatial arrangement of active sites to maximize the utilization efficiency, but also helps impart better stability and crystallinity of the materials. As a result, the optimized catalysts showed very impressive catalytic performance in terms of stereoselectivity and diastereoselectivity in Aldol and Diels–Alder reactions, which even surpassed the homogeneous analogues.

**Figure 8:**
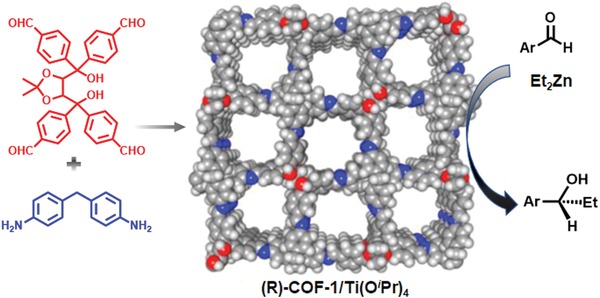
Synthetic scheme of CCOF and its application in the addition of diethylzinc to aromatic aldehydes after metalation with Ti(OiPr)_4_. Reproduced with permission.^39^ Copyright 2016, American Chemical Society.

In comparison with the chiral functionalities dangled on the framework, it is envisioned that those incorporated in the frameworks are expected to have a high level of synergism with the networks and thereby lead to superior performance. Cui and co‐workers demonstrated the construction of 2D COFs with chiral functionalities embedded into the framework by Schiff base condensation reactions of enantiopure tetraaldehydefunctionalized TADDOL (tetraaryl‐1,3‐dioxolane‐4,5‐dimethanols) and 4,4′‐diaminodiphenylmethane (**Figure** **8**).^39^ After being treated with Ti(O*^i^*Pr)_4_, this chiral COF showed superb performance in the asymmetric addition of diethylzinc to aldehydes, outperforming the corresponding homogeneous analogue (TADDOL/Ti) in terms of both activity and enantioselectivity. Moreover, due to their ordered microporous structures, obvious size selectivity toward reagents was observed in the COF‐based catalyst, given the fact that less than 5% conversions were detected with the CCOF/Ti catalyst in coronenyl transformation which was much lower than the 64% conversion obtained with homogeneous TADDOL/Ti. Later on, a 3D chiral COF based on TADDOL was reported by the same group through the condensation of tetra(4‐anilyl)methane with tetraaldehydefunctionalized TADDOL.^40^ The resultant COF material showed high performance in liquid chromatographic enantioseparation. Interestingly, the performance was further improved by the oxidization of the imine linkage into the amide form.

**Figure 9:**
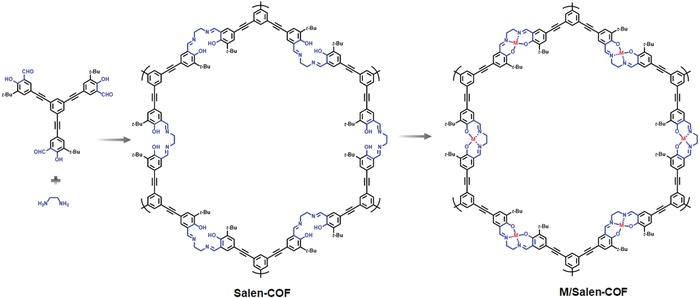
Synthetic scheme of Salen‐COF and M/Salen‐COF. Reproduced with permission.^41^ Copyright 2017, American Chemical Society.

The Salen unit represents one of the most important ligands in coordination chemistry. Wang and co‐workers demonstrated the first example of a Salen‐based COF material.^41^ By virtue of the similarity in the synthesis between Salen and imine‐based COFs, in this work, the construction of the COF structure and the functionalization with Salen moieties was realized in a single step (**Figure** **9**). Benefiting from the versatile coordination capability of Salen, a series of metallo‐Salen‐based COFs can be readily obtained via postsynthetic metalation (M/Salen‐COF). The PXRD patterns in these metalated Salen‐COFs indicated the maintained crystalline structures. Later, Cui and co‐workers developed this “kill two birds with one stone” strategy and two Salen‐based chiral COFs (CCOFs) were synthesized by imine‐condensations of enantiopure 1,2‐diaminocyclohexane and C_3_‐symmetric trisalicylaldehydes with or without the tert‐butyl group.^42^ To better induce the formation of Salen‐based CCOFs, Zn(OAc)_2_·2H_2_O was in situ introduced as a template during the COF synthesis. Given the relative liability of Zn—O/N bonds and the accessible channels as well as the robustness of the COF structure, a variety of metal species can be incorporated by postsynthetic metal exchange with negligible change in structure integrity. As a result, numerous efficient Salen‐based chiral catalysts can be designed on demand. Specifically, to catalyze the cyanation of aldehydes, V species were installed; to realize the asymmetric Diels–Alder reaction, Co species were immobilized; to fulfill a one‐step cascade reaction, in which epoxidation of the alkene was followed by ring opening of the epoxide to afford the amino alcohol, Cr and Mn species were simultaneously introduced. Moreover, the resultant catalysts can be readily recycled with retained catalytic performance in terms of both activity and selectivity for at least 5 times.

Photocatalytic water splitting represents a sustainable technique of producing clean energy from water by generating hydrogen. Lotsch and co‐workers showed how COFs possess all the necessary requirements needed to serve as an ideal platform for the design of photoactive catalysts. Specifically, using a triphenylarene platform, they show that the materials' photocatalytic water reduction can be readily engineered at the molecular level.^43^ Three triphenylarylaldehydes with the central aryl ring containing 0–3 nitrogen atoms were synthesized as building blocks to react with hydrazine to yield COF materials, N*x*‐COF, where *x* stands for the number of N in the central aryl ring of the precursor aldehydes. Progressively enhanced hydrogen evolution was observed with increasing nitrogen content in the frameworks, giving 23, 90, 438, and 1703 µmol h^−1^ after 8 h for N_0_‐COF, N_1_‐COF, N_2_‐COF, and N_3_‐COF, respectively. These results indicated about fourfold increase in hydrogen evolution with each substitution of C—H by N in the central aryl ring of the COF platform and N_3_‐COF rivaled the state‐of‐the‐art carbon nitride photocatalysts. To rationalize the observed trend and to provide insights into the mechanism underlined, using a combination of spectroscopic and theory calculation studies, it was revealed that the substitution of the C—H moiety with nitrogen atoms leads to a change in the dihedral angle between the central aryl ring and the peripheral phenyl rings, which in turn resulted in varied degrees of planarity in the platform and hence the stacking of COF layers. A decreased dihedral angle leads to improved crystallinity and layer registry, which facilitates exciton migration within the COF plane and thereby boosts the overall photocatalytic efficiency. Furthermore, a progressive decrease in electron density in the central aryl ring of the COF platform, as the number of nitrogen atoms increase from 0 to 3, results in a more stable form with the anion radical, which enhances the charge separation, thereby increasing the probability of successful electron migration to a nearby Pt cocatalyst.

**Figure 10:**
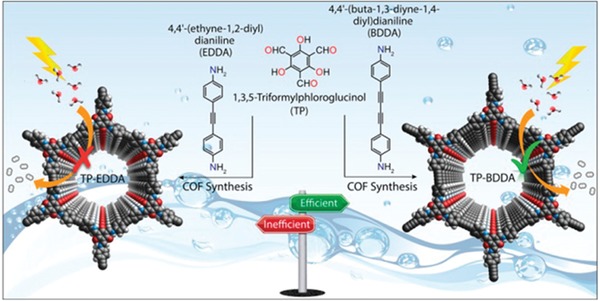
Synthetic scheme of TP‐EDDA and TP‐BDDA COFs and their use as photocatalysts for hydrogen generation from water. Adapted with permission.^44^ Copyright 2018, American Chemical Society.

Thomas and co‐workers also illustrated that the tunability of COF synthesis enables a sophisticated manipulation of the performance of the resultant catalysts. Two highly porous and chemically stable acetylene (—C≡C—) and diacetylene (—C≡C—C≡C—) functionalized β‐ketoenamine COFs were synthesized, which were than applied as photocatalysts for hydrogen generation from water using Pt as a cocatalyst (**Figure** **10**).^44^ It was shown that the diacetylene moieties have a profound effect as the diacetylene‐based COF outperformed the acetylene‐based COF in terms of photocatalytic activity by a factor of 6. These works highlight that COFs as a designer platform are very attractive to establish structure–property relationships.

In comparison with noble metal catalysts, developing efficient photocatalytic systems for hydrogen evolution using nonprecious‐metal‐based catalysts is more desirable. Lotsch and co‐workers developed a light‐induced proton reduction catalytic process by the combination of COFs as the molecularly defined photoabsorber with an earth‐abundant molecular cocatalyst, cobaloximes, for efficient hydrogen evolution.^45^ The resultant catalytic system was very efficient with a H_2_ evolution rate of 390 µmol g^−1^ h^−1^, which is 7.5 times higher than that of N_2_‐COF (52 µmol g^−1^ h^−1^) with Pt species under identical conditions. These results give a new insight for the development of efficient photocatalytic hydrogen evolution systems.

Photoelectrochemical water splitting is a very attractive way to generate hydrogen using renewable energy. Bein and co‐workers first explored COFs as photoelectrodes for such applications in the absence of both cocatalyst and sacrificial agent. The COF (BDT‐ETTA) was constructed by Schiff base reactions between 1,1′,2,2′‐tetra‐*p*‐aminophenylethylene (ETTA) and benzo[1,2‐b:4,5‐b′]‐dithiophene‐2,6‐dicarboxaldehyde (BDT).^46^ The remarkable stability of BDT‐ETTA in a wide range of pH aqueous solutions and strong absorption of visible light makes it a promising photoabsorber material. A great emphasis of this work was placed on the importance of the formation of an oriented BDT‐ETTA film to amplify the photoresponse of BDT and to maintain the long‐term stable performance.

**Figure 11:**
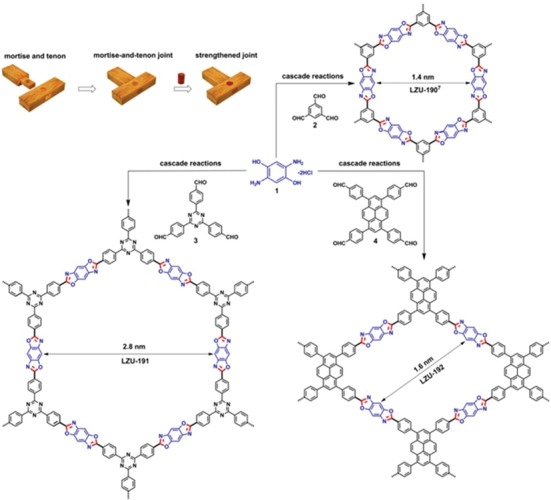
Strengthened mortise‐and‐tenon joints in COF synthesis via benzoxazole formation. Adapted with permission.^47^ Copyright 2018, American Chemical Society.

Developing a synthetic strategy to make the covalent joints both ultrastable and functional, Wang and co‐workers advanced a versatile strategy for synthesizing high performance COF‐based photocatalysts, whereby the formation of benzoxazole linkages not only stabilizes the COF structure but also decreases the optical bandgap and therefore increases the capability for visible‐light absorption. The benzoxazole‐linked COFs, LZU‐190, LZU‐191, and LZU‐192, were synthesized via the condensation of 1) 2,5‐diamino‐1,4‐benzenediol dihydrochloride with different aldehyde building blocks of 2) 1,3,5‐triformylbenzene 3) 2,4,6‐tris(4‐formylphenyl)‐1,3,5‐triazine, and 4) 1,3,6,8‐tetrakis(4‐formylphenyl)pyrene, respectively (**Figure** **11**).^47^ Due to the unique structure of the amine unit used, in which a hydroxyl group is placed at the ortho position of an amine group, the extended COF formations experienced a cascade reaction involving reversible imine bond formation followed by irreversible oxazole ring formation. This reversible/irreversible sequence could be ideal for the construction of robust COF structures. This not only enables self‐healing to yield high quality crystals but also reinforces the bond in the final material together with increasing light absorption. As a result of the irreversible formation of aromatic benzoxazole joints throughout the frameworks, the synthesized COFs exhibited superior structural stability compared with imine‐linked COFs. Such that, in the visible‐light‐driven oxidative hydroxylation of arylboronic acids to phenols, the resultant catalysts displayed excellent photoactivity and extraordinary recyclability (for at least 20 catalytic runs, each with a product yield of 99%).

In addition to serving as a platform for introducing photocatalytically active building units, COFs can also be used as a support to immobilize photoresponsive species. For example, Banerjee and co‐workers integrated a COF with photocatalytically active CdS nanoparticles for visible‐light‐driven hydrogen production.^48^ It was revealed that the π‐conjugated framework of COFs could not only stabilize the nanoparticles but also suppress the recombination of CdS photogenerated holes and electrons by facilitating an efficient charge transfer. As a result, the CdS–COF hybrids showed greatly enhanced photocatalytic H_2_ evolution as compared to the bulk CdS. For example, a 30‐fold augment was observed after introducing 10 wt% of the COF material during the hybrid synthesis relative to the bulk CdS.

COFs not only provide a platform for the incorporation of molecular catalysts, their ordered and customizable pore structures are promising templates for the confined growth of ultrafine metal nanoparticles. Zhang and co‐workers showed how COFs display the right combination of properties to serve as ideal host materials for size‐controlled synthesis of nanoparticles, as demonstrated by entrapping Pt and Pb nanoparticles onto a thioether‐containing COF.^49^ A set of control experiments revealed that the crystallinity of the COF support and the presence of thioether groups inside the cavities are critical for the size‐controlled synthesis of ultrafine NPs. In this work, with the aid of the evenly distributed thioether groups that have strong binding affinity toward noble metal species in the ordered framework structure, ultrafine Pt or Pd nanoparticles (1.7 ± 0.2 nm) with a narrow size distribution were obtained. The resultant catalysts showed excellent catalytic activity respectively in nitrophenol reduction and Suzuki–Miyaura coupling reaction under mild conditions and low catalyst loading. Benefiting from well‐isolated pore channels in COFs, preventing the entrapped NPs from aggregation, and the robustness of the COF framework under various reaction conditions, these catalysts are highly stable and readily recycled and reused without loss of their catalytic activities. Banerjee and co‐workers showed that COFs can be used as an alternative to address the aggregation of nanoparticles as demonstrated by the immobilization of gold nanoparticles on to a COF, TpPa‐1, which was developed by their group.[[qv: 9f]] Given the homogeneous dispersion and the strong interaction between the COF and the Au nanoparticles, the resultant catalyst, Au(0)@TpPa‐1, showed superior activity and recyclability for the nitrophenol reduction reaction.^50^

The development of cascade reaction processes by performing the consecutive reaction steps in one pot offered enormous economic advantages, given that costly intermediate separation and purification processes are avoided. To accomplish this, it is highly desirable to integrate different catalytically active sites within a single and recyclable material to facilitate one‐pot cascade catalysis.^51^

Considering the unique architectures and amenability of 2D COFs, they are expected to serve as an auspicious platform for integrating multiple components to bring into effect the cascade catalysis. We illustrated that a family of bifunctional COF materials can be obtained by partial metalation of a highly porous and chemically robust pyridine functionalized COF (COF‐TpPa‐Py).^52^ With Pd species present and by taking advantage of the base catalytic behavior of uncoordinated pyridine, catalysts contained two types of catalytic species, Pd/pyridine complex and pyridine, whereby the ratio of the two catalytic components can be readily adjusted by varying the Pd species loading amount. Catalytic evaluation results indicated these bifunctional COF materials exhibited excellent performance in the one‐pot cascade aerobic oxidation‐Knoevenagel condensation reactions to yield α,β‐unsaturated dinitriles from alcohols, far outperforming the corresponding homogeneous catalytic system as well as porous organic polymer‐based catalytic systems in terms of turnover frequency. These phenomena can be reasonably attributed to the combined features of the ordered porous structure, highly accessible active sites, and the site isolated manner of two catalytic components in the COF‐based catalyst.

Besides catalytic species being introduced after synthesis, the intrinsic activity of COFs can also be used for cascade reactions. Qiu and co‐workers demonstrated the construction of two new 3D COFs with both acidic and basic sites, resulted from the linkages boroxine and imine, respectively, and their application in acid/base bifunctional catalysis.^53^ Reacting tetrahedral TAA with 4‐formylphenylboronic acid or 2‐fluoro‐4‐formylphenylboronic acid, afforded two 3D bifunctional COFs named as DLCOF‐1 and DLCOF‐2, respectively. Due to the high crystallinity, both DLCOF‐1 and DLCOF‐2 showed high surface areas, giving 2259 and 2071 m^2^ g^−1^, respectively. To explore the catalytic potential of these COFs, acid–base catalyzed one‐pot cascade reactions, which involve acid catalyzed hydrolysis of the acetal followed by base catalyzed Knoevenagel condensation, were chosen. Catalytic results indicated that both COFs showed high activity and recyclability.

**Figure 12:**
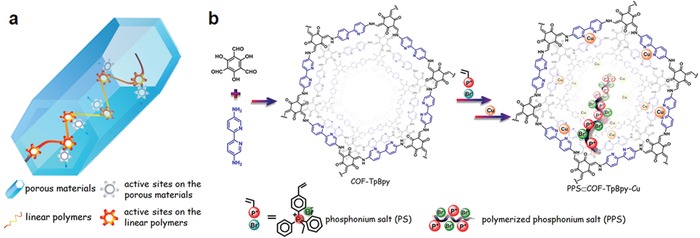
a) The concept of heterogeneous concerted catalysis between active sites on the porous materials and highly flexible linear polymers and b) schematic of PPS⊂COF‐TpBpy‐Cu synthesis and structures of COF‐TpBpy and PPS⊂COF‐TpBpy‐Cu.^54^ Copyright 2016, American Chemical Society.

The integration of multiple catalytically relevant functionalities into a single material is a concept often employed by biological catalysts and is an emerging strategy in heterogeneous and homogeneous catalysis. In an ideal scenario, the functional groups act cooperatively to enhance reactivity and/or selectivity. Nevertheless, challenges remain to design and prepare multifunctional catalysts featuring cooperative active sites, especially for heterogeneous catalysis, as the active sites spatially separated within the rigid framework are usually difficult to cooperate. We proposed a general and effective strategy for concerted heterogeneous catalysis as demonstrated by encapsulating catalytically active linear polymers within the channels of a COF bearing another type of catalytic component.^54^ As a proof‐of‐concept study, ionic polymers with halide anions were placed into a COF bearing Lewis acid sites via in situ polymerization (**Figure** **12**). In the cycloaddition reaction of CO_2_ with epoxides, the resultant composites exhibit significantly improved catalytic efficiency compared to individual ones. Also they clearly outperform the combination of the organic ionic compounds and the COF catalyst, a benchmark catalytic system. More importantly, the composite is stable and can be recycled for at least ten cycles with negligible loss in performance. The flexibility and movability of the catalytically active linear polymers facilitate the two types of catalytic species proximate with each other, which enables the double‐activation process. Furthermore, the locally enriched concentration of the active species in the composite catalysts results in high utilization efficiency, thereby leading to superior catalytic performance. This study is important because it affords an amenable route to bridge natural and artificial systems, given that the strategy presented herein requires neither particular types of porous materials nor linear polymers. It is also envisioned that the simple and effective method of fabrication of the composites, will promote the development of materials for a wide range of applications.

The exploration of new porous hybrid materials is of great importance for designing efficient catalytic materials. Zhang and co‐workers gave the first example, by integration of a photoactive MOF, NH_2_‐MIL‐68, and a COF material, which was grown on the exterior of the MOF crystals, a new type of MOF@COF core–shell hybrid material with high crystallinity and hierarchical pore structure, was synthesized.^55^ The resultant composite was very active in the visible‐light‐driven photocatalyst for the degradation of rhodamine B, giving a rate constant of 0.077 min^−1^, which was 1.4 times that of NH_2_‐MIL‐68. The authors ascribed this superior performance to the increased BET surface area together with the smaller bandgap of the composite compared to the pristine NH_2_‐MIL‐68.

Another example to this realm is metal doped core–shell MOFs@COFs, reported by Kim and co‐workers, as demonstrated by Pd loaded on the core–shell structure composed of NH_2_‐MIL‐125(Ti) and COF‐LZU1 (Pd/TiATA@LZU1).^56^ A combination of spectroscopy and control experiments revealed that a synergy exists among the three components with the metal elucidated to be the active center, the MOF core as an electron donor, and the COF shell as a mediator for electron transfer. As a result, the resultant composites exhibited excellent performance in hydrogenation of olefins, dehydrogenation of ammonia borane, as well as challenging liquid–gas tandem dehydrogenation and hydrogenation reactions.

### 2.3 Environmental Remediation

**Figure 13:**
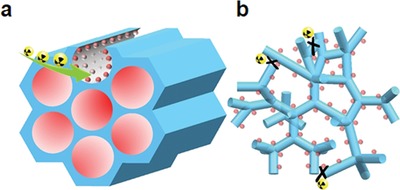
a) Schematic illustration of chelating groups in COF materials. The uniform pore morphology of the COFs leads to the functionalized material with unrestricted access of ions to all of the chelating sites. b) The functionalization of amorphous porous organic polymers, illustrating the blockage of narrow pore channels and bottlenecks. Pore‐blocking is likely to impede access of metal ions to the functional sites in amorphous porous oragnic polymers. Adapted with permission.^58^ Copyright 2018, John Wiley and Sons.

The potential of nuclear events demands continuous efforts for addressing such risk.^57^ Solid‐phase sorbents advanced in this area are expected to lead to more efficient remediation of contaminated effluent streams. To enhance the adsorption capacity, kinetics, and selectivity, the use of complexing functionalities has come into play to combat pollutions. However, a fraction of grafted chelating groups was found to be inaccessible due to the small and irregular pore structures of the amorphous host materials, thereby compromising their overall performance. By virtue of the unique structures and amenable synthesis of 2D COFs, we delineate important advances toward the implementation of COFs for construction of adsorbents.^58^ The periodic arrays of aligned chelating groups that are uncovered on the channels of the COFs facilitate ion entrapment and their cooperation in metal binding, which give new insight for the design of high performance sorbent materials (**Figure** **13**). The proof‐of‐concept study was illustrated by incorporating amidoxime groups on two newly designed COF materials linked by irreversible β‐ketoenamines for the sequestration of uranium. Due to the exceptional accessibility and unique orientation of chelating groups, the amidoxime functionalized COFs far outperform their corresponding amorphous analogues in terms of adsorption capacities, kinetics, and affinities in the mitigation of uranium in a range of contaminated water samples. Given the similarity in both surface area and amidoxime binding site content, it thereby can be concluded that the ordered pore structure of the COFs facilitates uranium uptake relative to the irregular structure of the amorphous counterparts. The resultant amidoxime chelating group‐laced COFs are capable of effective uranium removal from a wide range of water samples with outstanding uptake capacity (up to 408 mg g^−1^), and can rapidly diminish the uranium concentration down to 0.1 ppb level, which is far below the U.S. Environmental Protection Agency elemental limits for hazardous wastes and even drinking water standards (30 ppb). These materials also give promise for mining uranium from seawater for nuclear fuel production, showing an uptake capacity as high as 127 mg uranium per gram of adsorbent from spiked seawater. This work thereby puts forth a promising design strategy in optimizing the performance of adsorbents for environmental remediation.

**Figure 14:**
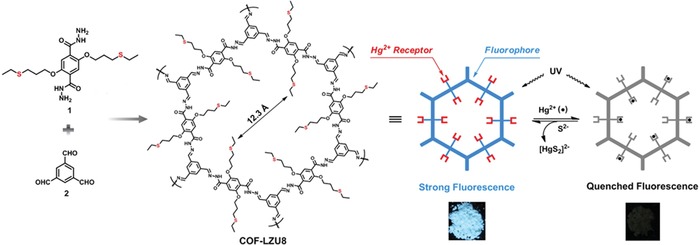
Synthesis of COF‐LZU8 via the cocondensation of **1** and **2** under solvothermal conditions. For clarity, the extended COF‐LZU8 structure is not shown. With the π‐conjugated framework as the fluorophore and the thioether groups as the Hg^2+^ receptor, the synthesized COF‐LZU8 was applied for both detection and removal of Hg^2+^. COF‐LZU8 exhibited strong fluorescence upon excitation at 390 nm. Upon the addition of Hg^2+^, the fluorescence of COF‐ LZU8 was effectively quenched. Photographs of COF‐LZU8 under a UV lamp (λ_ex_ = 365 nm) visualize the significant change in the fluorescence emission before (left) and after (right) the adsorption of Hg^2+^. Adapted with permission.^60^ Copyright 2016, American Chemical Society.

Heavy metal pollution, particularly mercury, is a persistent problem worldwide with grave public health consequences. Prompted by this critical need to mitigate heavy metal contamination and protect fresh water supplies, developing new materials for selective capture/detection of a target contaminant has long been a hot topic.^59^ Wang and co‐workers reported the first application of fluorescent COFs for selectively detecting and removing Hg ions, as demonstrated by a thioether‐functionalized hydrazine linked COF, COF‐LZU8, which was constructed by the condensation of **1** and **2** (**Figure** **14**).^60^ Due to the functionality being introduced de novo, the thioether groups are evenly‐and‐densely distributed in the regular 1D channels of the resultant COF, enabling fast mass transfer and thereby allowing for real‐time detection. Given the high affinity of thioether toward mercury, the COF can selectively capture Hg^2+^ from a variety of interference ions and thereby the realization of efficient detection with a detection limit as low as 25 ppb. Given these, the COF was then used as an adsorbent for mercury removal with an uptake capacity of around 300 mg g^−1^. Due to the robustness of the hydrazone linkage, the COF can be repeatedly used, showing great promise for real application. Jiang and co‐workers also showcased a thioether functionalized COF material (TAPB‐BMTTPA‐COF) by Schiff base condensation reaction between tris(4‐aminophenyl)benzene (TAPB) and 2,5‐bis(methylthio)terephthalaldehyde (BMTTPA) for mercury removal from aqueous solutions.^61^ Due to the increased sulfur species in the resultant COF material, TAPB‐BMTTPA‐COF showed a greatly improved mercury uptake capacity, giving 734 mg g^−1^, 2.5 times that of COF‐LZU8.

**Figure 15:**
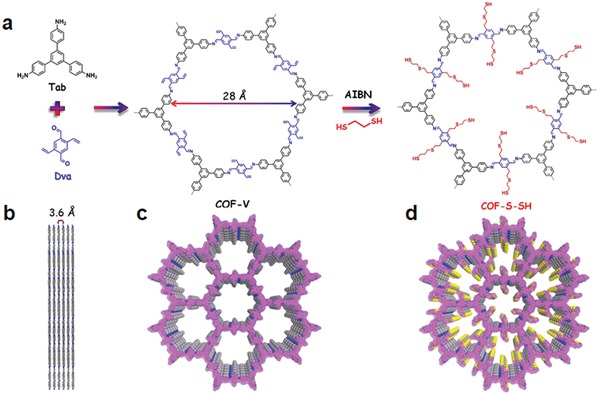
a) Synthetic scheme of COF‐V through the condensation of Tab (black) and Dva (blue) and representative channel‐wall engineering by thiol‐ene reaction with (COF‐S‐SH). b,c) Graphic view of the slipped AA stacking structure of COF‐V (blue, N; grey, C; hydrogen is omitted for clarity). d) Graphic view of COF‐S‐SH (blue, N; grey, C; yellow, S; hydrogen is omitted for clarity). Adapted with permission.^62^ Copyright 2017, American Chemical Society.

We also showed that 2D mesopore COFs possess all the traits to serve as a scaffold for decorating binding sites to create ideal adsorbents for toxic metal species decontamination, as illustrated by modifying sulfur derivatives on a newly designed vinyl‐functionalized mesoporous COF (COF‐V) via thiol‐ene “click” reaction (**Figure** **15**).^62^ Due to the high throughput of the thio‐ene transformation, more than 90% of the vinyl groups participated in the reaction with retained crystallinity and porosity. Representatively, the material (COF‐S‐SH), synthesized by treatment of COF‐V with 1,2‐ethanedithiol, displayed high efficiency in removing mercury from both aqueous solutions and the gas phase with outstanding uptake capacity (up to 1350 and 863 mg g^−1^ for Hg^2+^ and Hg^0^, respectively). Remarkably, COF‐S‐SH demonstrates an ultrahigh distribution coefficient value (*K*_d_) of 2.3 × 10^9^ mL g^−1^, placing it within striking distance of the all‐time mercury uptake record. This allows it to rapidly reduce the Hg^2+^ concentration from 5 ppm to less than 0.1 ppb, well below the acceptable limit in drinking water (2 ppb), even in the presence of a high concentration of interfering ions. Control experiments revealed that the flexibility of the chelating arms has a profound effect on the removal efficiency of the adsorbents. The highly flexible chelating arms are able to arrange in different conformations, ready for metal ions to adopt a favorable form, thereby increasing the affinity, which is reminiscent of that seen in biological systems and protein receptors. These findings suggest the merits of COFs in the design of heavy metal capture for environmental remediation. X‐ray absorption fine structure spectroscopic results revealed that each Hg is bound exclusively by two sulfur atoms via intramolecular cooperativity in COF‐S‐SH, further interpreting its excellent affinity.

Valtchev and co‐workers reported a strategy for preparation of a 3D carboxyl‐functionalized COF (3D‐COOH‐COF) through postsynthetic modification of a hydroxyl COF, and explored it for selective extraction of lanthanide ions.^63^ To synthesize 3D‐COOH‐COF, a 3D hydroxyl COF was synthesized by condensation of tetra(4‐formylphenyl)methane and 3,3′‐dihydroxybenzidine, followed by the reaction of hydroxyl groups with succinic anhydride. Sorption results revealed that 3D‐COOH‐COF showed high metal uptake capacities together with excellent adsorption selectivity for Nd^3+^ over Sr^2+^ and Fe^3+^.

The ion‐exchange process is widely applied in many industrial and household applications for purification, separation, and decontamination purposes. Qiu and co‐workers demonstrated the synthesis of two new 3D ionic COFs and their potential application for the removal of hazardous ions. The COF materials were obtained by the condensation of tetrakis(4‐formylphenyl)methane with diimidium bromide or ethidium bromide (EB), named as 3D‐ionic‐COF‐1 and 3D‐ionic‐COF‐2, respectively.^64^ After proving their crystallinity, porosity, and stability, their potential application for the removal of permanganate was investigated. Given the accessibility of ion‐exchange sites, these COFs showed fast removal kinetics, outperforming the representative materials, PVBTAH‐ZIF‐8 and LDHs, reported. More important, these COFs are highly stable and the ion‐exchange process is fully reversible, thus enabling multiuse with retained performance.

Trabolsi and co‐workers reported for the first time the use of the Zincke reaction to fabricate a viologen‐linked covalent organic network with controllable morphology by varying the polymerization conditions, as demonstrated by the reaction between 1,1′‐bis(2,4‐dinitrophenyl)‐[4,4′‐bipyridine]‐1,1′‐diium dichloride and TAPB.^65^ Given the porosity and cationic nature of the synthesized materials, they were explored for I_2_ capture. Sorption tests revealed that these materials not only could efficiently remove I_2_ from solution, but also I_2_ vapor.

Organic micropollutants are emerging contaminants in global freshwater supplies. These compounds cannot be removed effectively by wastewater treatment plants, and their negative effects on the environment and human health are now coming into focus. The development of new materials to help combat this emerging global problem is urgent. The high chemical stability of β‐ketoenamine COFs in a wide range of chemical conditions allows for the multistep postsynthetic modification to impart them with new properties while retaining the pore structure of the pristine COF material. These features suggest COFs of this type to be promising candidates for selective adsorption processes. Bein and co‐workers presented the first demonstration of a two‐step postsynthetic modification in a β‐ketoenamine COF,^66^ TpBD(NO_2_)_2_, reported by Banerjee and co‐workers.^66^ The nitro groups in TpBD(NO_2_)_2_ were first reduced to the primary amines followed up by an aminolysis reaction of acetic anhydride to form an amide bond to yield TpBD(NH_2_)_2_ and TpBD(NHCOCH_3_)_2_, respectively. To show the influence of functionalities on the removal efficiency, adsorptive removal of lactic acid was studied, given that it is a major precursor for biodegradable plastics. A great increase in affinity of the framework for lactic acid upon reduction of the nitro group to the amine was observed, giving uptake capacities of 2.5 and 6.6 wt%, respectively.

Given that numerous dye molecules are charged, using electrostatic interaction is an alternative for their removal. To create such interfaces, Jiang and co‐workers rationally designed an ionic building unit, 5,6‐bis(4‐formylbenzyl)‐1,3‐dimethyl‐benzimidazolium bromide (BFBIm). By condensation of BFBIm and PyTTA, a cationic COF material with a BET surface of 1532 m^2^ g^−1^ was obtained (PyTTA‐BFBIm‐iCOF).^67^ Different from the eclipsed AA‐stacking of most 2D COFs, PyTTA‐BFBIm‐iCOF showed the reverse slipped AA‐stacking mode distributes the benzimidazolium cationic centers on both sides of the pore walls, allowing for improved accessibility of the ionic moieties on the COF. Such that, the COF exhibited a very high negative charged methyl orange uptake capacity of 553 mg g^−1^, which was the highest among adsorbent materials reported at that time.

The tunable functionality of the pore surface of COFs together with their ordered pore channels can be exploited for the separation of molecules according to the combined contribution of size, charge, and functionality. To demonstrate this, Loh and co‐workers designed a salicylideneanilines‐based COF material (SA‐COF) by condensation of 1,3,5‐tris(4‐aminophenyl)benzene and TFP.^68^ The resultant COF possesses a relatively high BET surface of 1588 m^2^ g^−1^ with a narrow pore size centered at 1.43 nm. Interestingly, SA‐COF exhibits reversible proton tautomerism triggered by adsorption and desorption of water molecules. Given the ordered pore channel and multifunctional groups, SA‐COF was then explored as a versatile adsorbent for water soluble organic pollutant separation. It was found that SA‐COF showed obvious size‐dependent adsorption, as demonstrated that its binding affinity toward dye molecules declined with an increase in molecular size and could completely exclude molecules with dimensions larger than the pore size. In addition to this, the —OH and —NH moieties in SA‐COF can be deprotonated or protonated in basic or acidic conditions, respectively, showing a pH‐controllable ionicity. By virtue of this, SA‐COF can efficiently separate charged molecules. Furthermore, given the basicity of the N—H moiety in the trans‐keto form, SA‐COF can discriminate functional groups with different acidities, and it selectively bonded —OH over —NH_2_ groups under neutral conditions.

**Figure 16:**
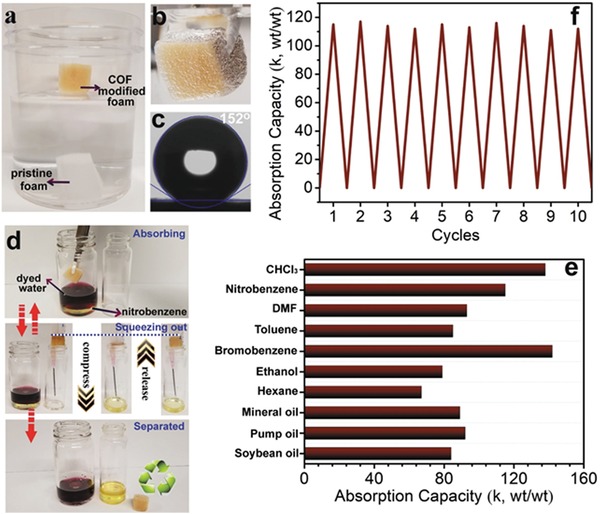
Superhydrophobic property and oil sorption performance tests. a) Photograph of COF‐VF@foam (yellow color) and melamine foam (white color) after being placed on water. b) Photograph of COF‐VF@foam immersed in water by a force. c) Water contact angle on the surface of COF‐VF@foam. d) COF‐VF@foam can separate underwater oil such as nitrobenzene from water. e) Absorption capacities of COF‐VF@foam for various organic solvents and oils, as indicated by weight gain. f) Weight gain during nitrobenzene absorption/squeezing cycles. Adapted with permission.^70^ Copyright 2018, Elsevier Inc.

Pollution by crude oil, petroleum products, and toxic organic solvents, which results in severe environmental and ecological problems, is a risk of growing concern. Worthy of note, oil spill cleanups amount to over 10 billion dollars annually. Remediation of these environmental issues involves the use of large amounts of adsorbents such as sand, activated carbons, or zeolites. However, the effectiveness of such adsorbents is often compromised by their affinity for moisture.^69^ Consequently, the search for highly hydrophobic porous materials to be used as a suitable stopgap of harmful organic spills has become of paramount importance. We contributed an effective strategy to impart superwettabilities on COFs as illustrated by chemical modification of the pore surface to confer them with superhydrophobicity (**Figure** **16**).^70^ Through judicious choice of fluorinated compounds and careful optimization of the postsynthetic modification conditions, the resultant COF exhibits superhydrophobic behavior, while retaining the porosity and crystallinity of the pristine COF. Significantly, due to the extreme water repellent properties of superhydrophobic surfaces, COF‐VF shows negligible water adsorption even at *P*/*P*_0_ up to 0.9 (less than 10 mg g^−1^), whereas it has a toluene uptake capacity exceeding 680 mg g^−1^ at *P*/*P*_0_ = 0.88. These results indicate that COF‐VF offers exceptional abilities to overcome the problems associated with the adsorption of harmful volatile organic compounds in humid environments. To add their applicability in the real world, experiments were designed to integrate them with substrates to increase their processability. We integrated COF‐VF with melamine foam and the resultant COF‐VF@foam shows highly efficient absorption of not only petroleum products, but also toxic solvents, such as chloroform and nitrobenzene, with an uptake capacity up to 142 times its own weight. Remarkably, the oil absorption kinetics of COF‐VF@foam was very rapid, reaching its saturated absorption capacity within 5 s with the captured oil readily removed by simple squeezing of the foam and the recovered COF‐VF@foam was reused without any loss in its performance, requiring no further treatment, thus suggesting that COF‐VF@foam is a promising candidate as a “suction skimmer.”

**Figure 17:**
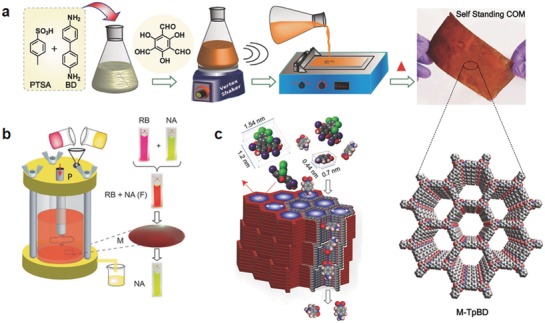
a) Schematic representation of the COMs (M‐TpBD) fabrication. b) Schematic of the nanofiltration assembly and selective molecular separation of nitroaniline (NA) from a mixture of NA and rose Bengal (RB), (“F” denotes feed and “P” for permeate). c) Schematic for molecular sieving mechanism through M‐TpBD. Reproduced with permission.^72^ Copyright 2017, John Wiley and Sons.

Nanofiltration membranes are widely used in wastewater treatment and recovery of valuable solutes. The use of crystalline porous materials to fabricate nanofiltration membranes is expected to address the issues of low permeability and selectivity encountered by traditional ones that are composed of amorphous polymers such as polyimide and poly(amide‐imide)based crosslinked polymers.^71^ In this context, Banerjee and co‐workers developed an eloquent methodology for fabrication of freestanding, porous, and crystalline membranes (COMs) composed of COFs via baking of building units in the presence of ptoluene sulfonic acid and water (**Figure** **17**a).^72^ Given that the COFs are connected by ketoenamine linkages, they exhibit excellent chemical resistance to a wide range of conditions including strong acid and base aqueous solutions, enabling their utilization in extreme conditions. In addition, due to the rigid aromatic framework structures, the resultant COMs are nonswelling. Furthermore, these freestanding COMs are flexible and continuous without any internal defects or cracks. With these advantages, these COMs exhibit high fluxes toward organic solvents, such as acetone and acetonitrile, with equivalent soluterejection performances, affording 278 L m^−2^ h^−1^ bar^−1^, which is 2.5‐fold higher than the existing polyamidenanofilmbased membranes (Figure 17b). Due to the ordered micropores, the COMs could completely reject the dye molecules with molecular dimensions above 1 nm, indicative of their potential toward wastewater treatment in textile/dye industries. In addition to this, these COMs can efficiently segregate bacteria from water to lower than its detection limit as demonstrated by *Escherichia coli* (Figure 17c). Given their high performance together with processable and cost effective properties, these COMs advanced in this work show great promise for real applications.

### 2.4 Sensors Based on COFs

Given that COFs show good photophysical properties, one might predict that they would act as efficient chemical sensors. Wang and co‐workers reported a newly designed 3D pyrene‐based fluorescent COF (3D‐Py‐COF) with a twofold interpenetrated pts topology.^73^ This COF was synthesized by the Schiff base reactions between tetra(*p*‐aminophenyl)methane and 1,3,6,8‐tetrakis(4‐formylphenyl) pyrene with a BET surface of 1290 m^2^ g^−1^ and microporosity centered at 0.59 nm. Due to the isolated imine‐functionalized pyrene units in the 3D network, 3D‐Py‐COF showed an intense yellow green luminescence, which was further explored for explosive detection. Using picric acid (PA) as the model, fluorescence of 3D‐Py‐COF was gradually quenched along with PA concentration increased in the suspension. A 75% quenching degree can be reached when the concentration of PA is 20 ppm, indicative of its high sensitivity to PA.

To address the concerns of the moderate electron delocalization and hydrolytic stability of imine‐linked COFs, which may impede their real application, Perepichka and co‐workers developed a new dynamic polymerization based on Michael addition‐elimination reactions of β‐ketoenols with amines for the preparation of COF materials.^74^ As a result of enhanced π‐conjugation, the bandgap (1.8–2.2 eV) in the resultant COFs is significantly reduced in comparison with imine‐linked COFs (0.2–0.3 eV), which also exhibit solid state luminescence and reversible electrochemical doping. The combination of the π‐conjugation with specific host–guest interactions within the pores of the resultant COF gives rise to a remarkable sensing behavior. To show their application in explosives detection, triacetone triperoxide (TATP) was chosen as a model compound, considering that it is a notoriously dangerous explosive, yet it remains a challenge to efficiently detect due to its relative chemical inertness. It was found that the fluorescence of the COFs could be successfully quenched by TATP, probably attributable to an oxidation of the enamine moiety in COFs by TATP, which is supported by their color change.

In many cases, due to the aggregated π‐stacked layers that resulted in luminescence concentration quenching and ineffective interaction with analytes, the bulk 2D COFs exhibit only moderate chemical sensing ability. To address these challenges, Baneriee and co‐workers exfoliated the bulk COF (TfpBDH) synthesized from 1,3,5‐tris(4‐formylphenyl)benzene (Tfp) and pyromellitic‐N,N′‐bisaminoimide (BDH), to produce covalent organic nanosheets (CONs) by the liquid phase exfoliation method.^75^ Due to the weakened π–π interactions, the resultant CONs show superior chemical sensing capabilities compared to the bulk COF. Specifically, the sensitivity of the CONs toward nitroaromatic analyte detection is up to tenfold higher than the bulk COF, even at a very low [10 × 5 (m)] analyte concentration, thereby showing potential for explosion chemical detection. Very interestingly, TfpBDH‐CONs exhibit a “turn‐off” detection in the dispersion state, but conversely exhibit a superior “turn‐on” detection capability for 2,4,6‐trinitrophenol in the solid state, promising for its application in visual detection of nitroaromatic compounds.

**Figure 18:**
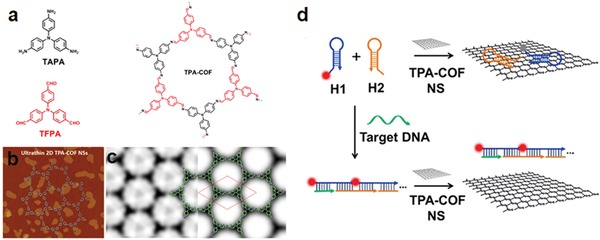
a) Schematic illustration of synthesis and extended hexagonal structure of the bulk TPA‐COF material. b) AFM image of TPA‐COF NSs with the thickness indicated. c) Left: Lattice‐averaged, P3‐symmetry imposed, and CTF‐corrected HRTEM image. Right: Simulated projected potential map with a point spread function width of 4 Å, in which the projected structural model in green and unit cell in red are embedded. d) Schematic illustration of a TPA‐COF NS‐based fluorescence sensor for detection of DNA. Reproduced with permission.^76^ Copyright 2017, American Chemical Society.

Not limited to small molecules, the covalent organic nanosheets can also be used as a novel sensing platform for DNA detection. Zhang and co‐workers showcased the first example with this respect.^76^ By elaborately designing and choosing the geometries of building units and their connection patterns, a novel [3 + 3] imine‐linked COF (TPA‐COF, **Figure** **18**), was synthesized by the condensation of tris(4‐aminophenyl)amine and tris(4‐formylphenyl)amine. Due to the flexibility of the building units and thereby weak interlayer stacking, the bulk COF can be readily exfoliated by sonication in an ethanol solution. The resultant ultrathin TPA‐COF NS shows excellent selectivity and high sensitivity in the detection of DNA by taking advantage of the strong π–π stacking interactions between the labeled fluorescent dye molecules and TPA‐COF NS, resulting in the fluorescence quenching of the dye. Later, Ajayaghosh and co‐workers prepared fluorescent cationic ultrathin 2D sheets EB‐TFP‐iCONs by self‐exfoliation of an ionic COF, EB‐TFP.^77^ Given the long and periodic phosphate backbone of DNA molecules, they can interact electrostatically with multiple numbers of positively charged EB‐TFP‐iCONs simultaneously leading to their strong restacking, accompanied by orange color emission. The DNA assisted reassembly of EB‐TFP‐iCONs resulted in a crystalline hybrid material, which in turn can be exploited as a fluorescent sensor material. The reassembly process was remarkably efficient with dsDNA, which enabled their discrimination from ssDNA, without tagging any fluorescent molecule.

### 2.5 COFs for Enzyme/Drug Uptake

Porous materials, such as mesoporous silica materials or MOFs, have long been studied and shown promise in drug delivery applications. Given their adjustable pores, excellent stability, and tunable compositions, COFs are expected to be effective materials for drug delivery. To show a proof‐of‐concept study for drug delivery applications, two new 3D COFs constructed by imidization between pyromellitic dianhydride and TAA or tetra(4‐aminophenyl)methane were rationally designed and named as PI‐COF‐4 and PI‐COF‐5, respectively.^78^ Ibuprofen (IBU) was chosen as a model drug due in part to it being widely used but processing a short biological half‐life (2 h). It was shown that both COFs exhibited high drug loading capacities as well as good release control. For both COFs, most of the IBU was released after about 6 days, and total delivery could reach ≈95% of the initial IBU loading. Moreover, the drug delivery in COFs can be adjusted by their pore size and geometry, given the fact that PI‐COF‐5 with the smaller pore size and interpenetrated structure shows a lower release rate in relation to that of PI‐COF‐4.

To realize a targeted drug delivery system and to increase the biocompatibility, Banerjee and co‐workers developed a promising strategy based on scalable salt‐mediated synthesis of COFs and subsequent functionality based sequential postsynthetic modifications to yield targeted materials.^79^ This postsynthetic modification resulted in simultaneous chemical delamination and functionalization to yield CONs. Specifically, postsynthetic modification on the hydroxyl functionalized COFs via ring‐opening of glycidol provided the necessary anchoring sites for conjugation of cellular targeting agents to the CONs for preferential delivery of a drug (5‐fluorouracil) to the cancer cells. As a result, sustained release of the drug from targeted CONs led to the death of cancer cells by apoptosis.

**Figure 19:**
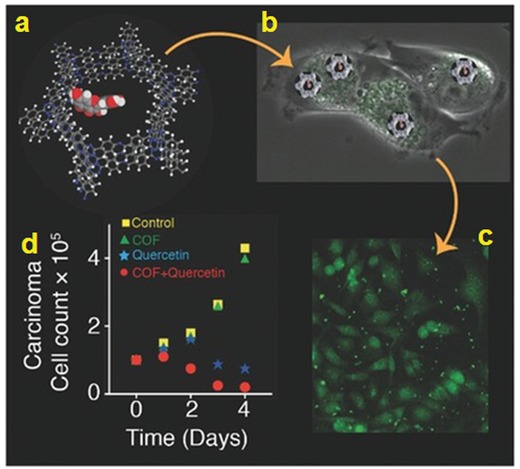
a) The view of the modeled COF pore showing the interaction of Quercetin with the pore wall. b) COF uptake by MDA‐MB‐231 carcinoma cells as seen in the merged phase contrast and green fluorescence channel image under the fluorescence microscope. c) Green fluorescence channel. d) Proliferation assay of the cancer cells treated with the COF (green triangles), Quercetin (blue stars), and Quercetin‐loaded COF (red dots) over a period of 4 days. The control experiment is shown as yellow squares. Adapted with permission.^80^ Copyright 2016, John Wiley and Sons.

Achieving better control of drug delivery requires knowledge of the interactions between the immobilized drug and its host material. Using a variety of spectroscopy techniques, Lotsch and co‐workers beautifully illustrated that the free electron pairs on the imine nitrogens of the COF are the main contributors for anchoring the guest molecules reversibly by H‐bonding interactions.^80^ Such interactions were further exploited for the targeted uptake and release of Quercetin (3,3′,4′,5,7‐pentahydroxyflavone) as a model drug. Proliferation control experiments using human breast carcinoma cells show that the drug encapsulated in the COF leads to a lower proliferation rate in comparison with that of direct drug administration, highlighting the potential of this class of materials used as a drug‐delivery vehicle (**Figure** **19**).

Coupling enzymes with solid supports affords a unique opportunity to shield them from deactivation, provides potential for recyclability, and increases the operational stability. Banerjee and co‐workers showed the first case of using COFs for enzyme encapsulation.^81^ A chemically stable hollow sphere COF with mesoporous walls synthesized by the Schiff base reactions between 2,5‐dihydroxyterephthalaldehyde (Dha; 21.6 mg) and 1,3,5‐tris(4‐aminophenyl)benzene (Tab) were used as the host material. Due to the strong intramolecular hydrogen bonding that locks the phenyl rings in one plane and protects the imine nitrogen from nucleophilic attack, the resultant COF material (COF‐DhaTab) showed high crystallinity with a BET surface area of 1480 m^2^ g^−1^ and narrow pore size distribution centered at 3.7 nm as well as excellent stability in a wide range of conditions including a phosphate buffer, a medium often used for enzyme loading. Considering the size constraints of the COF, trypsin with hydrodynamic size of around 3.8 nm was chosen for their studies. Successful immobilization of trypsin was verified by confocal laser scanning microscopy, revealing that most of the trypsin was immobilized in the mesoporous walls of the hollow spheres, given the fact that the hollow cavity of the sphere is nonfluorescent. Enzymatic assay revealed that around 60% of the activity of the free enzyme was retained after immobilization in the hydrolysis of *N*‐benzoyl‐L‐arginine‐4‐nitroanilide to *p*‐nitroaniline. Although the stability of the immobilized enzyme, such as tolerance to non‐natural conditions as well as recyclability, was not investigated, this work implies that COFs hold great promise for enzyme immobilization. This is expected to spur further endeavors on this aspect.

**Figure 20:**
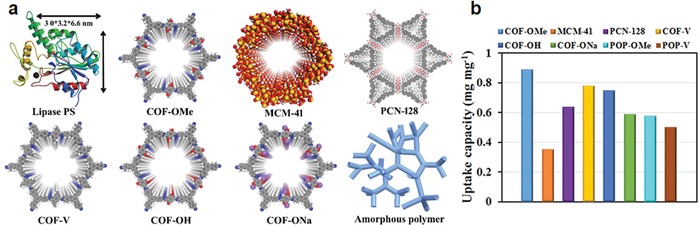
a) Graphic view of lipase PS and porous materials used for the immobilization of enzymes (blue, N; gray, C; red, O; white, H; yellow, Si; purple, Na). b) Enzyme uptake capacity of various porous materials after incubation in lipase PS solution (30 mg mL^−1^) for 6 h. Adapted with permission.^82^ Copyright 2018, American Chemical Society.

We have demonstrated, that due to the unique mesoporous structure and the tunable surface chemistry, COFs provided both high affinity for enzyme loading and a favored microenvironment that enhanced the enzymatic performance better than in other types of porous materials reported, as demonstrated by Lipase PS, a versatile type of enzyme (**Figure** **20**).^82^ This proven enhancement showed their utility with orders of magnitude higher catalytic activities compared to free enzymes and biocomposites made from other types of porous materials with similar porous structures and apertures, such as MOF (PCN‐128) and mesoporous silica (MCM‐41), as exemplified in the kinetic resolution of racemic 1‐phenylethanol with vinyl acetate. In addition, the COF‐based biocomposites also far outperform the composites made from their amorphous analogues, highlighting the important role of the long‐range order porous structure. Furthermore, it was found that coupling the enzyme with COFs also provided the additional benefits of recyclability and resistance to a wide range of environmental/industrial conditions, thereby giving great promise for providing “off the shelf” biocatalysts for synthetic transformations.

### 2.6 Miscellaneous Applications of COFs

#### 2.6.1 Photoconductivity

A distinctive aspect of 2D COFs is their long‐range π‐orbital overlap in the stacking direction, which facilitates charge carrier transport through the preorganized π‐pathways as well as gives rise to high exciton‐ and charge‐mobilities of interest for optoelectronic devices. The stacked π‐systems found in 2D COFs adopt an eclipsed fashion, facilitating their orbital interactions as well as providing aligned conduction pathways. In addition, their pores run parallel to the stacking direction, providing an opportunity to introduce complementary semiconductors. The resultant vertically aligned p–n junctions embody a morphology long thought to be ideal for photovoltaic performance. Moreover, unlike polymeric semiconductors, COFs generally show excellent thermal stability and do not undergo thermal phase transitions, which frequently require thermal or solvent annealing to attain morphologies suitable for reasonable photovoltaic performance.^83^

Jiang and co‐workers reported the first photoconductive COF (PPy‐COF, a hole‐transporting material) synthesized by self‐condensation of 2,7‐pyrene diboronic acid.^84^ Characterization results indicated that PPy‐COF was a super‐microporous crystalline macromolecule with an eclipsed alignment of polypyrene sheets. Photoconductivity observed in devices consisting of the COF powder sandwiched between Au and Al electrodes suggested long‐range exciton delocalization. A set of control experiments revealed that the quick response, together with the large on–off ratio could be reasonably attributed to the facilitated exciton migration and carrier transportation through the stacked pyrene moieties in PPy‐COF. In light of the unprecedented properties, this work represents an important step toward the use of COFs in optoelectronics and photovoltaics.

To improve the photoconductive performance, the crystallization of electron donors and acceptors into macroscopic heterojunctions with segregated donor and acceptor domain structures is highly pursued. 2D COFs provide an exceptional platform for precise control of the structure and its properties sincethe building blocks in 2D COFs are arranged in a perfect grid of repeating hexagons. In this context, Jiang and co‐workers demonstrated the first COF material that integrated donors and acceptors by cocrystallization of the building units bearing these moieties, as exemplified by triphenylene donor and benzothiadiazole acceptor.^85^ The aligned periodic independent pathways allowed for ambipolar electron and hole conductions and vertically ordered p–n heterojunctions with a broad donor–acceptor interface, leading to enhanced photoconductivity.

As an alternative, Bein and co‐workers proposed a supramolecular approach by spatially confining electron acceptors within the open channels of electron‐donating frameworks.^86^ Considering that special conformation of the monomer units is not required, this approach greatly expands the scope of donors and acceptors, whereby it is also applicable to the 0D molecules, such as fullerene derivatives, a class of widely utilized electron acceptors. To demonstrate this strategy, a new TT‐COF with high charge‐carrier mobilities was synthesized under solvothermal conditions by cocondensation of thieno[3,2‐b]thiophene‐2,5‐diyldiboronic acid and the polyol, HHTP. To introduce the fullerene electron acceptor [6,6]‐phenyl‐C_61_‐butyric acid methyl ester (PCBM), TT‐COF was soaked in a PCBM chlorobenzene solution. The resultant composite exhibited a significant photoresponse as well as supports efficient charge transfer within the lifetime of the excitons.

**Figure 21:**
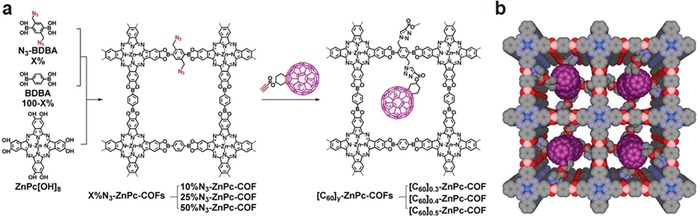
a) Schematic representation of converting open lattice structures into segregated donor–acceptor arrays. b) Top view of donor–acceptor COF with C_60_ (purple) integrated on the channel walls. Reproduced with permission.^87^ Copyright 2014, American Chemical Society.

Although this pore infiltration strategy is feasible, it encounters a problem with the fullerene elution from the channels. To tackle this concern, Jiang and co‐workers provided an alternative way toward organizing donors and acceptors into an ordered system, whereby the guest molecules were covalently anchored on the ordered pore channels (**Figure** **21**).^87^ To target this and to optimize the materials' performance, a three‐component condensation system of (2,3,9,10,16,17,23,24‐octahydroxyphthalocyaninato)zinc (ZnPc[OH]_8_) as vertices and 1,4‐phenylenediboronic acid (BDBA) and 2,5‐bis(azidomethyl)‐1,4‐phenylenediboronic acid (N_3_‐BDBA) at different molar ratios as edges under solvothermal conditions for the synthesis of a series of electron‐donating intermediate ZnPc‐COFs with adjustable contents of the azide units on the channel walls (*X*%N_3_‐ZnPc‐COFs, *X* = 0, 10, 25, and 50, N_3_ = azide unit) for further covalently anchoring C_60_. To show their potential application in photoenergy conversion, photoinduced electron transfer and charge separation were studied by means of time‐resolved electron spin resonance spectroscopy. It was revealed that the lifetime of the composites was governed by the delocalization of radical cations in the ZnPc columns, whereas the radical anions were localized at C_60_, thereby suggesting the cooperation between the COF skeleton and C_60_ anchored.

#### 2.6.2 Lithium Battery

**Figure 22:**
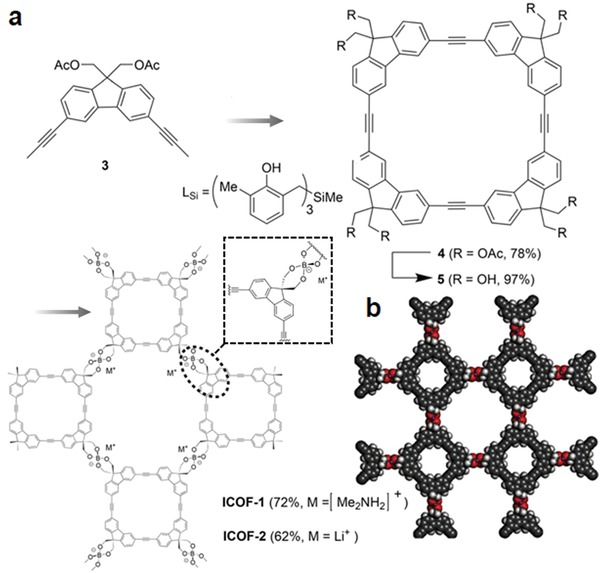
a) Synthesis of ICOF‐1 and ICOF‐2. b) Top view of proposed structural representations of ICOF‐2. Reproduced with permission.^88^ Copyright 2016, John Wiley and Sons.

Zhang and co‐workers reported the first example of ionic covalent organic frameworks (ICOFs) with sp^3^ hybridized boron anionic centers that were connected by transesterification of four diol groups in a D_4_‐symmetric macrocycle **5** and trimethyl borate by formation of spiroborate linkages (**Figure** **22**).^88^ The synthesized ICOFs possess the following two advantages: 1) due to the shape persistence of nanosized macrocycles, the resultant frameworks contain preexisting noncollapsible internal cavities; 2) given the robustness of the anionic framework, various types of catalytically active anions can be introduced, thereby allowing for tailoring of the material properties. In this paper, two novel ionic COFs with counter ions of (NMe_2_)^+^ and Li^+^ were synthesized and named as ICOF‐1 and ICOF‐2, respectively. N_2_ sorption isotherms collected at 77 K revealed that both of them are microporous materials with BET surface areas of 1022 and 1259 m^2^ g^−1^ for ICOF‐1 and ICOF‐2, respectively. However, due to the low quality and the complex PXRD data, the simulated crystal packing was not provided. Given the high content of accessible Li ions, ICOFs were thus explored as a Li‐conducting solid electrolyte. Advantageously being lightweight and possessing high thermal and chemical stability, a room‐temperature conductivity of 3.05 × 10^−5^ S cm^−1^ was observed. More interesting, being different to classic polymer electrolytes, which show nonlinear activation energy at higher temperatures, ICOF‐2 exhibits a linear characteristic of conductivity with temperature, reminiscent of ceramic solid conductors. The activation energy of ICOF‐2 was calculated to be 0.24 eV per atom, superior to those for typical solid‐state polymer electrolytes and even comparable to some of the state‐of‐the‐art crystalline solid electrolytes.

**Figure 23:**
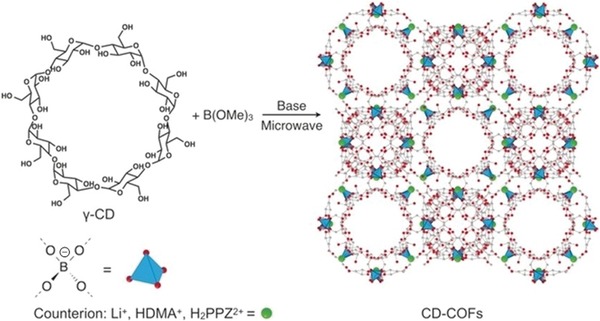
Condensation of γ‐CD and B(OMe)_3_ with LiOH, DMA, or PPZ under microwave conditions to afford CD‐COFs with different counterions. Adapted with permission.^89^ Copyright 2017, John Wiley and Sons.

Using cyclodextrin (CD) as the soft struts, Feng and co‐workers reported the construction of a 3D ionic COF through the reactions between γ‐CD and trimethyl borate, with the units joined via tetrakis(spiroborate) tetrahedra with various counter ions (CD‐COF, **Figure** **23**).^89^ Since the counter ions can be readily modulated and thereby the properties, this anionic open framework possesses numerous intriguing properties. For example, the COF with counter ions of Li^+^ hold great promise as a solid‐state electrolyte material with a high Li ion conductivity of up to 2.7 mS cm^−1^ at 30 °C and excellent long‐term Li ion stripping/plating stability.

Poly(ethylene oxide)‐based electrolytes have been heralded as promising materials for lithium ion batteries. However, as liquid electrolytes they also face issues such as leaking and safety. To solve these concerns encountered, the development of solid‐state polyelectrolytes is promising.^90^ Jiang and co‐workers reported the first example of polyelectrolyte COFs by implanting oligo(ethylene oxide) chains onto the channel walls to create a polyelectrolyte interface for the complexation of lithium ions.^91^ The Li^+^ ions infiltrated COF (Li@TPB‐BMTP‐COF) was synthesized by condensation of 1,3,5‐tri(4‐aminophenyl)benzene (TPB) with 2,5‐bis((2‐methoxyethoxy)methoxy)terephthalaldehyde (BMTP), followed by treatment with LiClO_4_ solution. The flexible oligo(ethylene oxide) chains on the channel walls facilitate the dissociation of the ionic bond of lithium salts upon complexation with lithium ions, thereby offering a pathway for ion conduction and promoting the ion transport through a low energy‐barrier vehicle mechanism. As a result, the ion conductivity was greatly enhanced by more than 3 orders of magnitude relative to that of ions across the bare nanochannels.

#### 2.6.3 Proton Conduction

The utilization of COFs in proton conduction had been impeded by their stability.^92^ This challenge was overcome by Banerjee and co‐workers.^93^ They developed a general strategy for synthesizing COFs that could maintain their structural integrity after being exposed to a strong acid (9 N HCl) and strong base (6 N NaOH), even upon isoreticulation and functionalization by construction of the chemically stable β‐ketoenamine‐linkage. To create a hydrogen‐bonding network in the COFs for proton transformation, the authors were inspired by the crystal structure formed by H_3_PO_4_ and 4‐aminoazobenzene in which H_3_PO_4_ is anchored on the azo (—N=N—) center by protonation, whereas the H_2_PO_4_‐anions are stabilized via H‐bonds. With these in mind, Banerjee and co‐workers synthesized an azo (—N=N—)‐functionalized COF (Tp‐Azo COF) by condensation of triformylphloroglucinol (Tp) and 4,4′‐azodianiline (Azo). After proving its stability against acid, a simple impregnation process was carried out to infiltrate phosphoric acid into the framework with an uptake capacity of 5.4 wt% (PA@TpAzo). Impressively, PA@TpAzo exhibited a great proton conductivity of 6.7 × 10^−5^ S cm^−1^ under anhydrous conditions. The azo units of Tp‐Azo COF were protonated and formed hydrogen‐bonding interactions with the H_2_PO_4_^−^ anion, which further interacted with free phosphoric acid molecules to form a hydrogen‐bonding network in the 1D channels, enabling the occurrence of self‐dissociation. The proton conductivity reached 9.9 × 10^−4^ S cm^−1^ under 98% relative humidity. These results imply that COFs offer an appealing platform for the construction of proton‐conducting systems for applications in fuel cells.

Given the 1D, ordered, mesoporous channels, and ultrastability of TPB‐DMTP‐COF, it is envisioned to enable a high loading of proton carriers and thereby improved proton conductivity. To use these advantages, Jiang and co‐workers employed this COF as a host material for loading proton carriers and imidazole and triazole were chosen as representative N‐heterocyclic proton carriers, yielding im@TPB‐DMTP‐COF and trz@TPBDMTP‐COF, respectively.^94^ Very high loading capacities of 155 and 180 wt% for imidazole and triazole were observed, close to their theoretical capacities of 163 and 186 wt%, respectively. Benefitting from the high proton carrier loading, the resultant composites exhibited excellent proton conductivities reaching 4.37 × 10^3^ S cm^−1^ at 130 °C, which is two orders of magnitude higher that of the state‐of‐the‐art imidazole‐loaded MOF.

**Figure 24:**
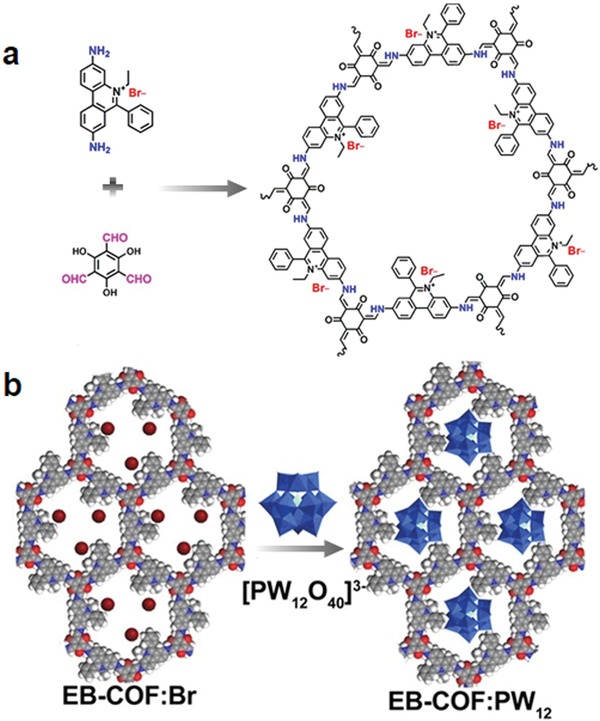
a) Schematic representation of the synthesis of EB‐COF:Br. b) Schematic of PW_12_O_40_^3‐^ doping in COF. Reproduced with permission.^95^ Copyright 2016, AmericanChemical Society.

Although, polyoxometalates show high performance in proton conduction, their high solubility represents a major hurdle that prevents them from real applications. To address this issue, Zhu and co‐workers developed a cationic COF to immobilize PW_12_O_40_^3−^ and then investigated its performance in proton conduction.^95^ The novel cationic COF material (EB‐COF:PW_12_) was obtained by combination of a cationic monomer, ethidium bromide (EB), with 1,3,5‐triformylphloroglucinol (EB‐COF:Br), followed by ion exchange with H_3_PW_12_O_40_ (**Figure** **24**). The proton conductivity improved along with the increase of relative humidity (RH), reaching 3.32 × 10^−3^ S cm^−1^ at RH (97%), which is two orders of magnitude higher than that of EB‐COF:Br. Under otherwise identical conditions, the physical mixture of H_3_PW_12_O_40_ and EB‐COF:Br only gave a value of 3.2 × 10^−5^ S cm^−1^. These results indicate the type and the dispersion degree of anions greatly affect the materials' proton conductive performance. It is worth mentioning that this is one of the best COF‐based proton conducting materials reported thus far.

#### 2.6.4 Energy Storage

**Figure 25:**
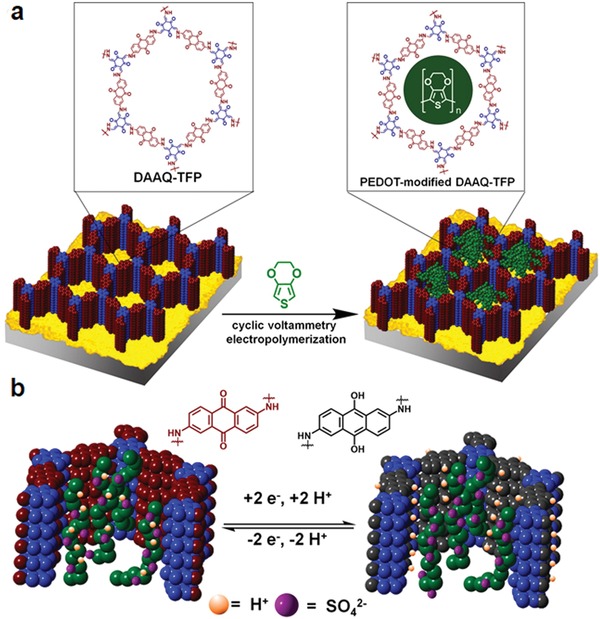
Incorporation of PEDOT within a DAAQ‐TFP COF film. a) Depiction of modification of DAAQ‐TFP films by electropolymerization of 3,4‐ethylenedioxythiophene (EDOT). Schematic depicts what may occur within one COF crystallite. b) Schematic of the cross‐section of a pore following the oxidation and reduction of the DAAQ moieties. Reproduced with permission.^97^ Copyright 2016, American Chemical Society.

2D COFs featuring ordered π‐columns and open nanochannels make them a promising candidate to be designed as energy storage materials. Excited by the fascinating H_2_Q/Q electrochemistry, it is envisioned that this chemistry could be incorporated in the COF backbone. Dichtel and co‐workers incorporated redox‐active 2,6‐diaminoanthraquinone (DAAQ) moieties into a 2D COF linked by β‐ketoenamines to confer outstanding hydrolytic stability.^96^ Due to its 2D layered architecture as well as increased capacitance relative to both its electroactive monomer and a COF lacking redox‐active groups, this material is the first COF to exhibit well‐defined, rapid redox processes. Despite the great promise of 2D COFs in optoelectronic and electrical energy storage devices, in particular for those constructed by redox‐active units as shown above, their low conductivity performance limited the devices to thin films of the active material (50–250 nm). Such that, operation is usually performed at slow charge/discharge rates, which restricts their application in high power devices. To address this challenge, Dichtel and co‐workers developed a strategy to enhance the electrical conductivity of a redox‐active 2D COF film by integrating a conductive polymer with the COF (**Figure** **25**).^97^ By taking advantage of the COF's ordered 1D channel, the poly(3,4‐ethylenedioxythiophene) (PEDOT) was threaded within the pores by electropolymerizing 3,4‐ethylenedioxythiophene (EDOT). The resultant PEDOT‐infiltrated COF films exhibited greatly improved electrochemical responses without compromising the accessibility to redox‐active groups on the pore wall, even for 1 µm thick COF films that otherwise provided poor electrochemical performance. Moreover, this composite could accommodate high charging rates (10–1600 C) and exhibited both an order of magnitude higher current response relative to unmodified films and stable capacitances for at least 10 000 cycles.

To improve the electrical conductivity, Jiang and co‐workers provided an alternative strategy. Stimulated by the excellent conductivity of 2D fully conjugated networks of graphene and related materials. They pioneered a topology‐directed reticular construction of crystalline sp^2^ carbon‐conjugated COF material by designing a C=C bond formation reaction.^98^ Through condensation reactions of tetrakis(4‐formylphenyl)pyrene and 1,4‐phenylenediacetonitrile in the presence of 4 m NaOH aqueous solution, the reactions were reversible, providing the self‐correction needed to form a crystalline material. Due to the extended π‐conjugated structure, the resultant COF is a semiconductor with a discrete bandgap of 1.9 eV. Nonetheless, its properties can be readily tailored. Chemical oxidation of this material with I_2_ greatly enhanced its conductivity, and the generated radicals confined on the pyrene centers imparted a high spin density and paramagnetism.

Using COFs as a designer platform, Jiang and co‐workers synthesized a series of redox‐active COFs for efficient energy storage by postsynthetic channel‐wall modification with free radical species.^99^ To investigate the effect of the density of the free radical species on the energy storage, a three component strategy was employed. Similar to their previous approach, COFs bearing ethynyl units were synthesized as intermediates, where the ethynyl group contents in the COFs were adjusted by another monomer. In this work, reactions of BPTA and DMTA at different molar ratios with nickel 5,10,15,20‐tetrakis(4′‐tetraphenylamino) porphyrin (NiP) gave rise to [HC≡C]*_X_*_%_‐NiP‐COF (*X* = 0, 50, and 100). The free radical species, as exemplified by (2,2,6,6‐tetramethylpiperidin‐1‐yl)oxyl (TEMPO), were then grafted by the click reaction. Obvious redox peaks were detected by cyclic voltammetry (CV) for the COFs containing the TEMPO moiety, whereas the pristine COFs did not show any reduction–oxidation response. These positive effects clearly confirmed the effectiveness of channel‐wall functionalization in introducing redox activity. According to the CV profiles, it was revealed that the output currents of these samples were augmented along with the density of the TEMPO functionality.

## 3 Perspective and Challenge

### 3.1 Structure

Unlike MOFs and supramolecular networks which are held together by bonds weak enough to be broken readily under their assembly conditions, allowing for rapid error correction and thereby permitting the growth of large single crystals, the vast majority of COFs reported thus far are produced as microcrystalline powders. Therefore, their structures are assigned by comparing the refined PXRD pattern with simulated patterns of eclipsed and staggered stacking arrangements, leading to controversy. Generally, it is well recognized that various kinds of out‐of‐plane disorder, such as random or poorly defined stacking sequences, and in‐plane defects, are prevalent.[[qv: 9k]] However, these offsets could hardly be confirmed experimentally for a long while, because peak broadening in powder samples is too large to differentiate between eclipsed and slightly offset structures. Previously, computational studies reveal that 2D COFs do not typically crystallize into fully eclipsed cofacial packing, as suggested by the simulated structure.^100^ It is likely that they adopt slightly offset structures to balance attractive forces with repulsive electrostatic interactions and the layers probably adopt random offsets from a vector normal to the stacking direction. However, no experimental evidence had been provided to prove this until very recently. An in‐depth study by electron diffraction and transmission electron microscopy gave direct evidences of the existence of grain boundaries and edge dislocations, which are likely generic to the in‐plane structure of 2D COFs, revealing a myriad of previously unknown or unverified structural features as well as confirming these calculations.

As shown above, even low quality COFs have shown preliminary promise for catalysis, water purification, storing electricity, and many more. Once developed further, higher‐quality samples of these materials will enable these applications to be explored more fully. Synthesizing single‐crystal COFs represents a longstanding challenge in material science. Previous attempts to make single crystalline COFs have been thwarted, in part because the formation of bonds between subunits is essentially irreversible, whereby crystallization cannot easily be tamed. This dilemma had not been over crossed until 2013, Wuest and co‐workers successfully isolated high quality covalent organic networks by nitroso self‐addition, allowing structures of the resultant materials to be determined in detail by single‐crystal X‐ray diffraction.^101^ This strategy is generally applicable. A series of 3D covalent single crystalline networks, NPN‐1, NPN‐2, and NPN‐3, was synthesized from oxidation polymerization of 1) nitroso compounds of tetrakis(4‐nitrosophenyl)methane, 2) tetrakis(4‐nitrosophenyl)silane, and 3) 1,3,5,7‐tetrakis(4‐nitrosophenyl)adamantine, respectively. However, due to the weak azodioxy bonds, these materials are unstable to solvent removal. The guest‐free samples did not exhibit permanent porosity and crystallinity, as established by N_2_ gas adsorption isotherms and powder X‐ray diffraction, respectively.

**Figure 26:**
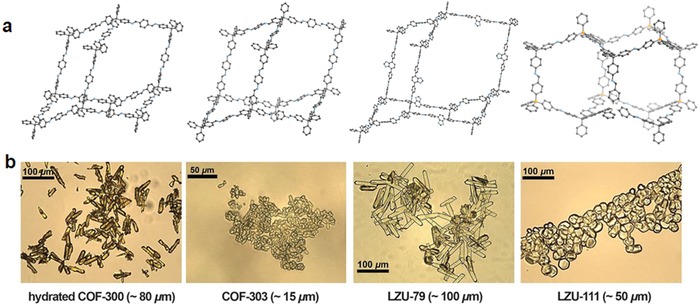
a) Structures and b) optical microscopy images of COF‐300, COF‐303, LZU‐79, and LZU‐111. Reproduced with permission.^102^ Copyright 2018, American Association for the Advancement of Science.

Very recently, a general procedure to grow large single crystals of 3D imine‐based COFs was developed (**Figure** **26**).^102^ To increase the reversibility of imine bond formation and dissociation and thereby allowing for efficient self‐correction of defects, the authors ingeniously adopted an imine‐exchange strategy involving the use of aniline as a modulator to slow the nucleation for the growing high‐quality crystals. For the first time, three stable, porous, single‐crystal 3D COFs with sizes up to 100 µm were isolated. The exceptional quality of the crystals allowed collection of single‐crystal X‐ray diffraction data with resolution comparable to small discrete molecules, leading to unambiguous solution and precise anisotropic refinement. Such that, the unresolved questions pertaining to the structure and guest molecules therein, such as the degree of interpenetration in imine‐based COF‐300 and the arrangement of water guests in the hydrated form of COF‐300, were clearly answered. Many other aspects, such as reversed imine connectivity, linker disorder, and uncommon topology can be deciphered, which is difficult without single crystals if not impossible.

**Figure 27:**
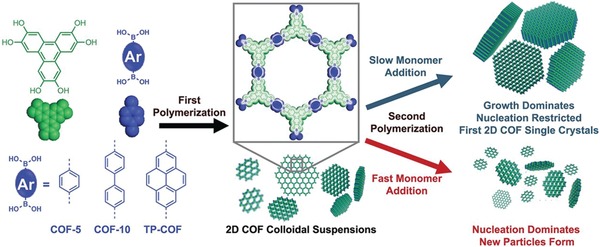
Schematic of controlled 2D polymerization. A two‐step seeded growth approach provides 2D COF single crystals. When HHTP and a linear bis(boronic acid) monomer are condensed in a solvent mixture containing CH_3_CN, crystalline 2D COF nanoparticles are formed as stable colloidal suspensions. These nanoparticles are enlarged in a second polymerization step in which the monomers are added to the solution slowly. If the monomers are added more quickly, their concentration increases above a critical nucleation threshold, which leads to uncontrolled nucleation and smaller average particle size. Adapted with permission.^103^ Copyright 2018, American Association for the Advancement of Science.

Nevertheless, the above developed two strategies were not generalized to 2D COFs. Almost at the same time as the publication of the 3D imine‐based COFs, Dichtel and co‐workers reported boronate ester‐linked 2D COFs isolated as discrete particles comprised of single crystalline domains with sizes ranging from 500 nm to 1.5 µm (**Figure** **27**).^103^ Different from using a modulator to control the crystal formation, a two‐step approach that separates the nucleation and growth processes was employed. Specifically, the scientists first grow small particle “seeds” to which they slowly add more of the building blocks, under carefully controlled conditions. The slow addition is necessary to allow the building blocks to add to the seeds instead of creating new seeds. Electron microscope results verified that the particles are individual and not aggregated and are perfectly uniform throughout the entire structure. To show these 2D COF samples have superior properties in comparison with their polycrystalline counterparts, a transient adsorption study was carried out, showing 1000‐fold improved signal quality and evidence for exciton delocalization at length scales in these high‐quality samples relative to the corresponding polycrystals. Another distinguished property exclusively emerged in high‐quality crystals is that energy can move throughout the structure after it absorbs light, which may be useful in solar energy conversion. Despite these successes, the development of single crystalline 2D COFs that are suitable for single‐crystal X‐ray diffraction, particularly those constructed by stable bonds, is still in the portfolio of researchers and such materials will answer the questions pertaining to the structure.

### 3.2 Processing

COFs are typically synthesized as powders under solvothermal conditions. The limited utility of these forms precludes many applications for COFs. For example, 2D COFs incorporate functional p‐electron systems into ordered structures ideally suited for optoelectronic devices. However, as unprocessable powders, these materials cannot be interfaced reliably to electrodes or incorporated into devices to harness or even quantify these properties. COF films offer an extraordinary opportunity to organize and orient functional p‐electron systems into robust periodic structures predictably through chemical synthesis. The growth of thin COF films with control over the film thickness, morphology, and crystallinity is highly desirable for the utilization of COFs in diverse applications. Dichtel and co‐workers introduced the first oriented 2D layered COF films, as demonstrated by COF‐5 and a Ni phthalocyanine‐1,4‐phenylenebis(boronic acid) (PBBA) COF, on substrate‐supported single layer graphene (SLG).^104^ The use of the graphene layer is expected to provide a suitable interface to favor the COF growth through π‐interactions on the surface. They also extended this strategy to COFs with greater pore sizes by synthesizing a family of phthalocyanine based COFs with pore size up to 4.4 nm as oriented thin films on substrate‐supported SLG.^105^ Later, Bein and co‐workers employed a similar synthetic procedure to integrate a new mesoporous electron‐donor covalent organic framework (BDT‐COF) synthesized by condensation of a benzodithiophene‐containing diboronic acid (BDTBA) and HHTP with different polycrystalline surfaces.^106^ The highly porous thin BDT‐COF films were then infiltrated with soluble fullerene derivatives, to obtain an interpenetrated electron‐donor/acceptor host–guest system. To study the electron transfer capabilities between the host and the guest, photoluminescence quenching experiments were performed, showing that the photoluminescence of the BDT‐COF film was quenched to a large extent by the hosted acceptors and thereby indicating the occurrence of charge transfer.

However, considering that the above methods employed for growing thin COF films are based on reactive precursor solutions under solvothermal conditions, COFs were formed as both an insoluble powder and as a continuous film on the substrate surface, challenges such as scalability, yield, and morphology control still remain. Alternative synthetic routes to form COFs are highly desirable. Bein and co‐workers ingeniously translate the concept of steam‐assisted conversion to the synthesis of a series of 2D COF films at room temperature.^107^ Taking the well‐known COF‐5 as an example, COF precursors dissolved in a polar solvent mixture of acetone and ethanol were drop‐casted on a clean glass substrate. The resultant material was then transferred into a desiccator along with a small vessel containing mesitylene and dioxane at a volume ratio of 1:1, a typical solvent system for synthesizing COF‐5. A complete conversion of the drop‐cast precursor solution into the final COF was achieved within 72 h at room temperature. Moreover, this novel method provides precise control of the film thickness ranging from a few hundred nanometers to several micrometers by either reducing the droplet volume or diluting the precursor solution, and allows for the realization of smooth and homogeneous coatings over large sample areas, even on different substrates. It is anticipated that this method will be of particular interest for fragile building blocks that do not survive the vigorous conditions typically used in solvothermal synthesis procedures. Wan and co‐workers also developed a self‐limiting solid–vapor interface reaction strategy to fabricate highly ordered surface COFs.^108^ Unlike the strategy demonstrated above, to better control the condensation and to minimize the unwanted formation of disordered oligomers, two precursors were physically separated. Precursor A, preloaded onto the substrate through drop casting, and precursor B were separately transferred in a closed reactor. By heating the reactor to a certain temperature, precursor B will vaporize and then land on the surface covered with precursor A, whereby the growth is controlled by the gas phase concentration of precursor B. Accordingly, the covalent bond was formed at the solid–vapor interface, leading to the growth of high quality surface covalent organic frameworks (SCOFs). Given the easy operation, this strategy is anticipated to be a general protocol for other types of bicomponent chemical reactions, not limited to Schiff‐base reaction as demonstrated in this contribution.

In addition to growing thin films, a surface deposition strategy has also been used for integrating COFs with other substrates to extend their application. For example, Yan and co‐workers applied a chiral COF based on tartaric acid as stationary phases for chiral separation, which were in situ integrated with substrates to synthesize chiral COF‐bound capillary columns.^109^ With a density of chiral centers aligned on the ordered 1D channel, using these as stationary phases gave high resolution performance. For some compounds, they even outperformed the commercial chiral capillary columns such as β‐DEX 225 and Cyclosil B. Although promising, concerns mentioned in the case of growing thin COF films based on reactive precursor solutions are all existent here and also are encountered when we grow COF‐VF onto melamine foam.^70^

Considering that it is uncommon for a material to be usable in applications as synthesized, they are typically processed into a specific form, such as a pellet, a thin polymer membrane, or a surface deposited film. Processability is therefore an important functional property that is often ignored, at least in the early discovery phase for a new material. Simple, scalable, and controllable processing routes with few steps are therefore desirable, irrespective of material type.

### 3.3 Synthesis

To have an impact on real applications, porous materials must be scalable. The first COF synthesis was performed under solvothermal reaction conditions, and this method to date is still the most often applied approach for COF formation. Thereby, the COF precursors are placed in a pyrex tube together with the desired solvent or solvent mixture and catalysts or modulators and then the tube is flash frozen at 77 K, evacuated, and flame‐sealed. The reaction mixture in these tubes can be heated above the normal boiling point of the solvent and the closed system also ensures that H_2_O is available to maintain reversible conditions, allowing for an increased solubility of the precursors and improved kinetics as well as structures for self‐correction. Although the solvothermal synthesis pathway in many cases leads to satisfactory results in terms of quality of the products, it also has some weaknesses. The most important could be the difficult transferability to industrial applications, as scale‐up is challenging and the reaction rates observed in many COF syntheses are very slow (normally 3 days). The search for alternative green, safe, and efficient technologies is one of the priorities in current scientific research. With this objective, some promising synthetic approaches were developed.

#### 3.3.1 Microwave‐Assisted Synthesis

Cooper and co‐workers showed that microwave synthesis could offer a highly convenient method for the rapid production of COFs, as demonstrated by COF‐5 and COF‐102.^110^ It greatly increases the crystallization rate by a factor of 200 with comparable physical properties as that prepared by solvothermal synthesis.

#### 3.3.2 Solid‐State Conversion

For some β‐ketoenamine COFs, mechanical grinding of the COF building units was found to yield the same COF structures as that known from solvothermal reactions. However, the synthesized COFs were less crystalline compared to the solvothermally synthesized batches and processed rather low BET surface areas of below 100 m^2^ g^−1^.^111^ Inspired by this, a vapor assisted solid‐state approach for COF synthesis was developed, whereby the finely ground COF linkers were transferred to a sealed container to be exposed to solvent vapor under heating. Improved crystal quality was achieved, yet still cannot compare with the solvothermal pathway.^112^

#### 3.3.3 Organic Terracotta Process

**Figure 28:**
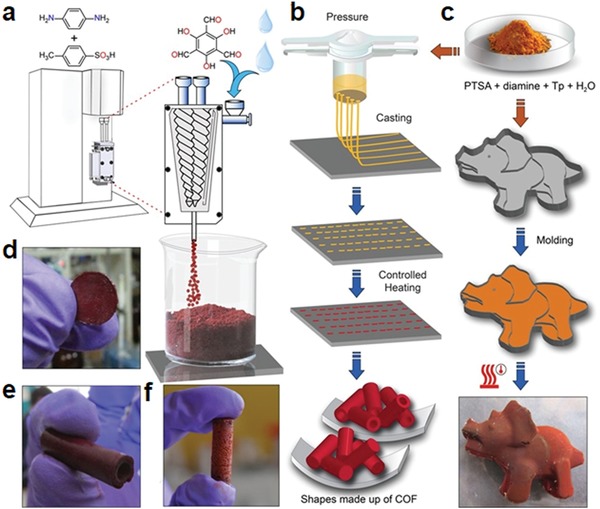
a) COF synthesis by extrusion. b) Schematic representation of COF‐bead fabrication using the terracotta technique. c) Schematic representation of COF processing into sculpture via the organic terracotta process. d–f) Digital photographs of COF membrane, hollow tube, and cylinder, respectively. Adapted with permission.^113^ Copyright 2017, American Chemical Society.

To increase the surface area and crystallinity of “solid‐state conversion,” Banerjee and co‐workers developed a liquid‐assisted grinding method, where a catalytic amount of liquid was introduced to the COF precursors during grinding.^113^ Specifically, the diamine linker was ground with *p*‐toluenesulfonic acid and subsequent addition of 1,3,5‐triformylphloroglucinol (Tp) and water, the ketoenamine COFs were obtained in high quality (BET surface area up to 3100 m^2^ g^−1^) after heating to 170 °C for 1 min. This strategy overcomes the long‐term synthetic hurdles of solvothermal synthesis as well as low surface area of the resultant COFs encountered by “solid‐state conversion.” Interestingly, the ground mixture had the consistency of dough and could be processed into various shapes without any additional binder, hence enabling the fabrication of COF sculptures via this “terracotta process” (**Figure** **28**).

#### 3.3.4 Room Temperature and Ambient Pressure Synthesis

Dichtel and co‐workers developed a metal‐triflate catalyzed room temperature synthesis of imine COFs that led to COFs with BET surface areas superior to the solvothermally synthesized frameworks. High quality COF materials with BET surface areas up to 2175 m^2^ g^−1^ can be achieved within 10 min.^114^ This method has also been extended to prepare large‐area, continuous COF films with controllable thickness.^115^ Fang and co‐workers successfully synthesized a series of 3D COFs under ambient conditions using ionic liquids as media within 12 h, which is substantially shorter than those from the traditional solvothermal method (3–7 days).^116^ However, expensive reagents such as metal‐triflate catalysts or ionic liquids were involved, which is undesirable for large‐scale synthesis.

#### 3.3.5 Vapor‐Assisted Conversion

This synthetic pathway was developed by Bein and co‐workers and the corresponding work has been introduced previously as ref. 107.

#### 3.3.6 Room Temperature Batch and Continuous Flow Synthesis

Zhao and co‐workers pioneered the room temperature continuous flow synthesis of COFs exemplified by COF‐LZU1, with an extremely high space‐time yield of 703 kg m^−3^ day^−1^.^117^ Dichtel and co‐workers obtained high quality 2D COF thin films under continuous flow conditions. However, there are prerequisites for this synthetic approach including good monomer solubility and strong π interactions serving as driving forces for the crystallization.^118^

COFs have shown preliminary promise for catalysis, environmental remediation, energy storage, and many more. Although progress has been made, this field of research is still in its infancy. Looking forward, a future direction would be to fully make use of construction principles for COFs to precisely integrate building blocks to achieve predesigned compositions, components, and functions. To find greater use at an industrial scale, COFs must be scalable and must satisfy multiple functional criteria such as long‐term stability, high performance, and processability, all within a viable cost envelope.

## Conflict of Interest

The authors declare no conflict of interest.
